# *Drosophila* Tachykininergic Neurons Modulate the Activity of Two Groups of Receptor-Expressing Neurons to Regulate Aggressive Tone

**DOI:** 10.1523/JNEUROSCI.1734-22.2023

**Published:** 2023-05-10

**Authors:** Margot P. Wohl, Jett Liu, Kenta Asahina

**Affiliations:** ^1^Salk Institute for Biological Studies, La Jolla, California 92037; ^2^Neuroscience Graduate Program, University of California, San Diego, La Jolla, California 92093

**Keywords:** aggression, *Drosophila*, G-protein-coupled receptor, neuromodulation, neuropeptide, tachykinin

## Abstract

Neuropeptides influence animal behaviors through complex molecular and cellular mechanisms, the physiological and behavioral effects of which are difficult to predict solely from synaptic connectivity. Many neuropeptides can activate multiple receptors, whose ligand affinity and downstream signaling cascades are often different from one another. Although we know that the diverse pharmacological characteristics of neuropeptide receptors form the basis of unique neuromodulatory effects on distinct downstream cells, it remains unclear exactly how different receptors shape the downstream activity patterns triggered by a single neuronal neuropeptide source. Here, we uncovered two separate downstream targets that are differentially modulated by tachykinin, an aggression-promoting neuropeptide in *Drosophila*. Tachykinin from a single male-specific neuronal type recruits two separate downstream groups of neurons. One downstream group, synaptically connected to the tachykinergic neurons, expresses the receptor *TkR86C* and is necessary for aggression. Here, tachykinin supports cholinergic excitatory synaptic transmission between the tachykinergic and *TkR86C* downstream neurons. The other downstream group expresses the *TkR99D* receptor and is recruited primarily when tachykinin is overexpressed in the source neurons. Differential activity patterns in the two groups of downstream neurons correlate with levels of male aggression triggered by the tachykininergic neurons. These findings highlight how the amount of neuropeptide released from a small number of neurons can reshape the activity patterns of multiple downstream neuronal populations. Our results lay the foundation for further investigations into the neurophysiological mechanism by which a neuropeptide controls complex behaviors.

**SIGNIFICANCE STATEMENT** Neuropeptides control a variety of innate behaviors, including social behaviors, in both animals and humans. Unlike fast-acting neurotransmitters, neuropeptides can elicit distinct physiological responses in different downstream neurons. How such diverse physiological effects coordinate complex social interactions remains unknown. This study uncovers the first *in vivo* example of a neuropeptisde from a single neuronal source eliciting distinct physiological responses in multiple downstream neurons that express different neuropeptide receptors. Understanding the unique motif of neuropeptidergic modulation, which may not be easily predicted from a synaptic connectivity map, can help elucidate how neuropeptides orchestrate complex behaviors by modulating multiple target neurons simultaneously.

## Introduction

Neuromodulation plays an important role in controlling ethologically important survival behaviors (LeDoux, 2012; Castro and Bruchas, 2019), including social behaviors (Insel, 2010). Neuropeptides are a major class of neuromodulator and are important for a variety of innate behaviors, such as feeding, fear and stress responses, sleep, and reproduction (Nässel and Winther, 2010; Castro and Bruchas, 2019). Because of its behavioral relevance, the neuropeptidergic system has been a major target for the development of effective therapeutics (Hökfelt et al., 2003; Holmes et al., 2003; Griebel and Holsboer, 2012). Neuropeptides that are released into the circulatory system act as neurohormones, but growing evidence indicates that neuropeptides can also locally modulate specific target neurons (Salio et al., 2006; Nässel, 2009; van den Pol, 2012; Nusbaum et al., 2017). For instance, several neuropeptides alter the physiology of a critical circuit node only during a specific hunger state, which ultimately changes the dynamics of the behavior-controlling circuit (Krashes et al., 2009; Ko et al., 2015; Oh et al., 2019). Flexibility in release sites and cotransmission with fast-acting neurotransmitters mean that neuropeptides can have an impact on the physiology of neurons beyond that predicted by the connectome (Salio et al., 2006; Nässel, 2009; Bargmann, 2012; Marder, 2012; van den Pol, 2012; Nusbaum et al., 2017). Indeed, findings in invertebrate nervous systems, such as those of crustaceans and nematodes, indicate that the behaviorally relevant chemoconnectomes of neuromodulators are dynamic and multifunctional (Flavell et al., 2013; Leinwand and Chalasani, 2013; Nusbaum et al., 2017). Although specific neuropeptidergic cell populations are often important for controlling survival behaviors in both vertebrates and invertebrates, how a single source of neuropeptides can coordinate the activity of multiple behaviorally relevant target neurons remains poorly understood.

In this study, we characterized the impacts of peptidergic neuromodulation in microcircuits that control intermale aggression in the fruit fly, *Drosophila melanogaster*. The male-specific Tk-GAL4^FruM^ neurons are known to promote aggressive behavior in part by releasing the neuropeptide tachykinin (Asahina et al., 2014; Wohl et al., 2020). We created new genetic alleles that label tachykinin receptor-expressing neurons to probe how tachykinin modulates targets downstream of Tk-GAL4^FruM^ neurons. Functional calcium imaging across the brain revealed two distinct, spatially restricted subsets of downstream neurons, each expressing a different *Drosophila* tachykinin receptor (*TkR86C* or *TkR99D*). Neurons that express *TkR86C* receive both cholinergic and tachykinergic inputs from Tk-GAL4^FruM^ neurons. A lack of tachykinin input diminished the ability of Tk-GAL4^FruM^ neurons to activate *TkR86C*-expressing neurons, suggesting that the function of this specific tachykinin input is to maintain the strength of cholinergic neurotransmission between the two neuronal populations. By contrast, neurons that express *TkR99D* are activated only when an excess amount of tachykinin is released from Tk-GAL4^FruM^ neurons. The differential impact of tachykinin on these two downstream populations correlates with the level of aggression promoted by optogenetic activation of Tk-GAL4^FruM^ neurons. Collectively, our results identify a receptor-based neuronal mechanism of tachykininergic neuromodulation. Distinct activation dynamics between *TkR86C* and *TkR99D* neurons provides insight into how neuropeptides can act to control a complex behavior and reshape the physiological dynamics of target circuits. Our findings underscore the significance of functional connectivity based on peptide–receptor relationships (the chemoconnectome).

## Materials and Methods

### Fly strains

Table 1 contains the complete genotypes of *Drosophila* strains used in each figure.

**Table 1.** Complete genotypes of *Drosophila* strains used in this study

Figure	Section	Abbreviated genotype	Complete genotype
Figure 1	*B*, *E*, *F*, *I*, *J*, *K*	Tk-GAL4^F^*^ru^*^M^ neurons→CsChrimson:tdTomato in Tk mutant	*w*, *Tk-GAL4^1^*; *Otd-nls:FLPo* in attP40 / +; *20*×*UAS*>*myr:TopHAT2*>*CsChrimson:tdTomato* in attP2, Δ*Tk^1^ / ΔTk^1^*
	*C*, *E*, *G*, *I*, *J*, *K*, *L*, *N*	Tk-GAL4^F^*^ru^*^M^ neurons→CsChrimson:tdTomato in Tk +/+ (WT)	*w*, *Tk-GAL4^1^*; *Otd-nls:FLPo* in attP40 / +; *20*×*UAS*>*myr:TopHAT2*>*CsChrimson:tdTomato* in attP2 / +
	*D*, *E*, *H*, *I*, *J*, *K*, *M*, *N*	Tk-GAL4^F^*^ru^*^M^ neurons→CsChrimson:tdTomato + UAS-Tk	*w*, *Tk-GAL4^1^*; *Otd-nls:FLPo* in attP40 / *10*×*UAS-Tk* in attP40; *20*×*UAS*>*myr:TopHAT2*>*CsChrimson:tdTomato* in attP2 / +
Figure 2	*A_1-3_*, *C–E*	Tk-GAL4^F^*^ru^*^M^ neurons (GFP), Postsynaptic marker (DenMark)	*w*, *Tk-GAL4^1^*; *tub*>*GAL80*> / *pJFRC-UAS-TLN:mCherry* in attP40; *fru^FLP^* / *pJFRC81-10*×*UAS-IVS-Syn21-GFP-p10* in attP2
	*B_1-3_*, *F–H*	Tk-GAL4^F^*^ru^*^M^ neurons (GFP), Presynaptic marker (Syt:GFP)	*w*, *Tk-GAL4^1^*; *tub*>*GAL80*> / *10*×*UAS-IVS-myr:tdTomato* in attP40; *fru^FLP^* / *5*×*UAS-IVS-Syt:GFP* in Su(Hw)attP1
	*I_1, 2_, J_1, 2_*	Tk-GAL4^F^*^ru^*^M^ neurons→CsChrimson:tdTomato	*w*, *Tk-GAL4^1^*; + / +; *20*×*UAS*>*myr:TopHAT2*>*CsChrimson:tdTomato* in attP2 / *fru^FLP^*
	*M*	TkR86C^L^*^ex^*^A^→GCaMP6f	*w*, *Tk-GAL4^1^*; *13*×*LexAop2-IVS-GCaMP6f-p10* in su(Hw)attP5 / +; *20*×*UAS*>*myr:TopHAT2*>*CsChrimson:tdTomato* in attP2, *TkR86C^LexA^, fru^FLP^* / *13*×*LexAop2-IVS-GCaMP6f-p10* in su(Hw)attP1
	*N_1, 2_, O, P_1, 2_, Q*	Tk-GAL4^F^*^ru^*^M^ neurons→CsChrimson:tdTomato, TkR86C^L^*^ex^*^A^→GCaMP6f	
Figure 3	*B_1, 2_, C_1-5_, D_1-5_, E, F*		
Figure 4	*A_1-4_, B–G*	Tk-GAL4^F^*^ru^*^M^ neurons→CsChrimson:tdTomato, *Trans-*Tango→GFP	*w, Tk-GAL4^1^; trans-Tango* in attP40, *tub*>*GAL80*> / + ; *20*×*UAS-IVS-CsChrimson:tdTomato* in attP2 / *QUAS-mCD8:GFP, fru^FLP^*
	*I_1, 2_, J, K*, *O*, *P*	Tk-GAL4^F^*^ru^*^M^→CsChrimson:tdTomato, *Trans-*Tango→GCaMP6f	*w, Tk-GAL4^1^; trans-Tango* in attP40, *tub*>*GAL80*> / 15×*QUAS-IVS-GCaMP6f-p10* in Su(Hw)attP5 ; *20*×*UAS-IVS-CsChrimson:tdTomato* in attP2 / 15×*QUAS-IVS-GCaMP6f-p10* in Su(Hw)attP1, *fru^FLP^*
	*M*, *N*, *P*	Tk-GAL4^F^*^ru^*^M^ neurons→CsChrimson:tdTomato, TkR86C^L^*^ex^*^A^→GCaMP6f	*w*, *Tk-GAL4^1^*; *13*×*LexAop2-IVS-GCaMP6f-p10* in su(Hw)attP5 / +; *20*×*UAS*>*myr:TopHAT2*>*CsChrimson:tdTomato* in attP2, *TkR86C^LexA^, fru^FLP^* / *13*×*LexAop2-IVS-GCaMP6f-p10* in su(Hw)attP1
Figure 5	*A_1-3_*	Tk-GAL4^1^→nls:tdTomato *ChAT*→nls:GFP	*w, Tk-GAL4^1^; 10×UAS-IVS-nls::tdTomato* in VK00022 / + ; *13*×*LexAop2-IVS-nls::GFP* in VK00040/*ChAT-LexA:QFAD0*
	*B_1-3_*	Tk-GAL4^1^→nls:tdTomato *VGlut*→nls:GFP	*w, Tk-GAL4^1^; 10×UAS-IVS-nls::tdTomato* in VK00022 / *VGlut-LexA:QFAD2* ; *13*×*LexAop2-IVS-nls::GFP* in VK00040 / +
	*C_1-3_*	Tk-GAL4^1^→nls:tdTomato Gad1→nls:GFP	*w, Tk-GAL4^1^; 10×UAS-IVS-nls::tdTomato* in VK00022 / + ; *13*×*LexAop2-IVS-nls::GFP* in VK00040/*Gad1-LexA:QFAD2*
	*E_1, 2_, F_1, 2_*	Tk-GAL4^F^*^ru^*^M^ neurons→CsChrimson:tdTomato, TkR86C^L^*^ex^*^A^→GCaMP6f	*w*, *Tk-GAL4^1^*; *13*×*LexAop2-IVS-GCaMP6f-p10* in su(Hw)attP5 / +; *20*×*UAS*>*myr:TopHAT2*>*CsChrimson:tdTomato* in attP2, *TkR86C^LexA^, fru^FLP^* / *13*×*LexAop2-IVS-GCaMP6f-p10* in su(Hw)attP1
Figure 6	*C–E*, *G*	*Tk-GAL4^1^*, *UAS*>*STOP*>*CsChrimson*, *Otd-nls:FLPo*, *TkR86C^LexA^*, *LexAop2-shibire^ts^*	*w*, *Tk-GAL4^1^*; *Otd-nls:FLPo* in attP40 / +; *20*×*UAS*>*myr:TopHAT2*>*CsChrimson:tdTomato* in attP2, *TkR86C^LexA^*/*13*×*LexAop2-IVS-Syn21-shibire^ts^* in VK00005
		*Tk-GAL4^1^*, *UAS*>*STOP*>*CsChrimson*, *Otd-nls:FLPo*, *LexAop2-shibire^ts^*	*w*, *Tk-GAL4^1^*; *Otd-nls:FLPo* in attP40 / +; *20*×*UAS*>*myr:TopHAT2*>*CsChrimson:tdTomato* in attP2 /*13*×*LexAop2-IVS-Syn21-shibire^ts^* in VK00005
		*Tk-GAL4^1^*, *UAS*>*STOP*>*CsChrimson*, *Otd-nls:FLPo*, *TkR86C^LexA^*	*w*, *Tk-GAL4^1^*; *Otd-nls:FLPo* in attP40 / +; *20*×*UAS*>*myr:TopHAT2*>*CsChrimson:tdTomato* in attP2, *TkR86C^LexA^*/ +
		*UAS*>*STOP*>*CsChrimson*, *Otd-nls:FLPo*, *TkR86C^LexA^*, *LexAop2-shibire^ts^*	*w*; *Otd-nls:FLPo* in attP40 / +; *20*×*UAS*>*myr:TopHAT2*>*CsChrimson:tdTomato* in attP2, *TkR86C^LexA^*/*13*×*LexAop2-IVS-Syn21-shibire^ts^* in VK00005
		*Tk-GAL4^1^*, *UAS*>*STOP*>*CsChrimson*, *TkR86C^LexA^*, *LexAop2-shibire^ts^*	*w*, *Tk-GAL4^1^*; + / +; *20*×*UAS*>*myr:TopHAT2*>*CsChrimson:tdTomato* in attP2, *TkR86C^LexA^*/*13*×*LexAop2-IVS-Syn21-shibire^ts^* in VK00005
Figure 7	*D_1, 2_*	Tk-GAL4^F^*^ru^*^M^ neurons→CsChrimson:tdTomato, TkR86C^L^*^ex^*^A^ neurons→GCaMP6f in Tk mutant	*w*, *Tk-GAL4^1^*; *13*×*LexAop2-IVS-GCaMP6f-p10* in su(Hw)attP5 / +; *20*×*UAS*>*myr:TopHAT2*>*CsChrimson:tdTomato* in attP2, *TkR86C^LexA^, ΔTk^1^* / *13*×*LexAop2-IVS-GCaMP6f-p10* in su(Hw)attP1, *fru^FLP^, ΔTk^1^*
	*E_1, 2_*	Tk-GAL4^F^*^ru^*^M^ neurons→CsChrimson:tdTomato, TkR86C^L^*^ex^*^A^ neurons→GCaMP6f in Tk +/+ (WT)	*w*, *Tk-GAL4^1^*; *13*×*LexAop2-IVS-GCaMP6f-p10* in su(Hw)attP5 / +; *20*×*UAS*>*myr:TopHAT2*>*CsChrimson:tdTomato* in attP2, *TkR86C^LexA^* / *13*×*LexAop2-IVS-GCaMP6f-p10* in su(Hw)attP1, *fru^FLP^*
	*F_1, 2_*	Tk-GAL4^F^*^ru^*^M^ neurons→CsChrimson:tdTomato, TkR86C^L^*^ex^*^A^ neurons→GCaMP6f in + UAS-Tk	*w*, *Tk-GAL4^1^*; *13*×*LexAop2-IVS-GCaMP6f-p10* in su(Hw)attP5 / *10*×*UAS-Tk* in attP40; *20*×*UAS*>*myr:TopHAT2*>*CsChrimson:tdTomato* in attP2, *TkR86C^LexA^* / *13*×*LexAop2-IVS-GCaMP6f-p10* in su(Hw)attP1, *fru^FLP^*
Figure 8	*A_1-3_, D_1-3_*, *E*, *H*	Tk-GAL4^F^*^ru^*^M^ neurons→CsChrimson:tdTomato, TkR86C^L^*^ex^*^A^ neurons→GCaMP6f in Tk mutant	*w*, *Tk-GAL4^1^*; *13*×*LexAop2-IVS-GCaMP6f-p10* in su(Hw)attP5 / +; *20*×*UAS*>*myr:TopHAT2*>*CsChrimson:tdTomato* in attP2, *TkR86C^LexA^, ΔTk^1^* / *13*×*LexAop2-IVS-GCaMP6f-p10* in su(Hw)attP1, *fru^FLP^, ΔTk^1^*
	*B_1-3_, D_1-3_*, *F*, *H*, *K*	Tk-GAL4^F^*^ru^*^M^ neurons→CsChrimson:tdTomato, TkR86C^L^*^ex^*^A^ neurons→GCaMP6f in Tk +/+ (WT)	*w*, *Tk-GAL4^1^*; *13*×*LexAop2-IVS-GCaMP6f-p10* in su(Hw)attP5 / +; *20*×*UAS*>*myr:TopHAT2*>*CsChrimson:tdTomato* in attP2, *TkR86C^LexA^* / *13*×*LexAop2-IVS-GCaMP6f-p10* in su(Hw)attP1, *fru^FLP^*
	*C_1-3_, D_1-3_*, *G*, *H*, *K*	Tk-GAL4^F^*^ru^*^M^ neurons→CsChrimson:tdTomato, TkR86C^L^*^ex^*^A^ neurons→GCaMP6f in + UAS-Tk	*w*, *Tk-GAL4^1^*; *13*×*LexAop2-IVS-GCaMP6f-p10* in su(Hw)attP5 / *10*×*UAS-Tk* in attP40; *20*×*UAS*>*myr:TopHAT2*>*CsChrimson:tdTomato* in attP2, *TkR86C^LexA^* / *13*×*LexAop2-IVS-GCaMP6f-p10* in su(Hw)attP1, *fru^FLP^*
	*I*	TkR86C^L^*^ex^*^A^→TkR86C:HA	*w*; *13*×*LexAop2-IVS-TkR86C:HA* in attP40 / +; *TkR86C^LexA^* / +
	*J*	*UAS*>*STOP*>*CsChrimson*, *fru^FLP^*, *TkR86C^LexA^*, *LexAop2-TkR86C*	*w*; *13*×*LexAop2-IVS-TkR86C* in attP40 / +; *20*×*UAS*>*myr:TopHAT2*>*CsChrimson:tdTomato* in attP2, *TkR86C^LexA^* / *fru^FLP^*
		*Tk-GAL4^1^*, *fru^FLP^*, *TkR86C^LexA^*, *LexAop2-TkR86C*	*w*, *Tk-GAL4^1^*; *13*×*LexAop2-IVS-TkR86C* in attP40 / +; *TkR86C^LexA^* / *fru^FLP^*
		*Tk-GAL4^1^*, *UAS*>*STOP*>*CsChrimson*, *TkR86C^LexA^*, *LexAop2-TkR86C*	*w*, *Tk-GAL4^1^*; *13*×*LexAop2-IVS-TkR86C* in attP40 / +; *20*×*UAS*>*myr:TopHAT2*>*CsChrimson:tdTomato* in attP2, *TkR86C^LexA^* / +
		*Tk-GAL4^1^*, *UAS*>*STOP*>*CsChrimson*, *fru^FLP^*, *LexAop2-TkR86C*	*w*, *Tk-GAL4^1^*; *13*×*LexAop2-IVS-TkR86C* in attP40 / +; *20*×*UAS*>*myr:TopHAT2*>*CsChrimson:tdTomato* in attP2 / *fru^FLP^*
		*Tk-GAL4^1^*, *UAS*>*STOP*>*CsChrimson*, *fru^FLP^*, *TkR86C^LexA^*	*w*, *Tk-GAL4^1^*; + / +; *20*×*UAS*>*myr:TopHAT2*>*CsChrimson:tdTomato* in attP2, *TkR86C^LexA^* / *fru^FLP^*
		*Tk-GAL4^1^*, *UAS*>*STOP*>*CsChrimson*, *fru^FLP^*, *TkR86C^LexA^*, *LexAop2-TkR86C*	*w*, *Tk-GAL4^1^*; *13*×*LexAop2-IVS-TkR86C* in attP40 / +; *20*×*UAS*>*myr:TopHAT2*>*CsChrimson:tdTomato* in attP2, *TkR86C^LexA^* / *fru^FLP^*
Figure 9	*C, D_1-4_, E, F*	Tk-GAL4^F^*^ru^*^M^ neurons→CsChrimson:tdTomato, TkR99D^L^*^ex^*^A^ neurons→GCaMP6f	*w*, *Tk-GAL4^1^*; *13*×*LexAop2-IVS-GCaMP6f-p10* in su(Hw)attP5 / +; *20*×*UAS*>*myr:TopHAT2*>*CsChrimson:tdTomato* in attP2, *TkR99D^LexA^* / *13*×*LexAop2-IVS-GCaMP6f-p10* in su(Hw)attP1, *fru^FLP^*
Figure 10	*C_1, 2_, D, E*, *I*, *J*		
	*F_1, 2_, G, H, I, J*	Tk-GAL4^F^*^ru^*^M^ neurons→CsChrimson:tdTomato, TkR99D^L^*^ex^*^A^ neurons→GCaMP6f in + UAS-Tk	*w*, *Tk-GAL4^1^*; *13*×*LexAop2-IVS-GCaMP6f-p10* in su(Hw)attP5 / *10*×*UAS-Tk* in attP40; *20*×*UAS*>*myr:TopHAT2*>*CsChrimson:tdTomato* in attP2, *TkR99D^LexA^* / *13*×*LexAop2-IVS-GCaMP6f-p10* in su(Hw)attP1, *fru^FLP^*
Figure 11	*A_1, 2_*	TkR99D^L^*^ex^*^A^ ∩ fru^FLP^→CsChrimson:tdTomato	*w*; + / +*; TkR99D^LexA^, 13×UAS>myr:TopHAT2>CsChrimson:tdTomato* in attP2 / *fru^FLP^*
	*C*, *D*	TkR99D^L^*^ex^*^A^, LexAop2>stop>CsChrimson, fru^FLP^	
	*D*	LexAop2>stop>CsChrimson, fru^FLP^	*w*; + / +*; 13*×*UAS*>*myr:TopHAT2*>*CsChrimson:tdTomato* in attP2 / *fru^FLP^*
		TkR99D^L^*^ex^*^A^, fru^FLP^	*w*; + / +*; TkR99D^LexA^* / *fru^FLP^*
		TkR99D^L^*^ex^*^A^, LexAop2>stop>CsChrimson	*w*; + / +*; TkR99D^LexA^, 13×UAS>myr:TopHAT2>CsChrimson:tdTomato* in attP2 / +

Genotypes are listed in the order of appearance. “>” represents the flippase recognition target (FRT).

*Tk-GAL4^1^* (RRID:BDSC_51975), *Otd-nls:FLPo* (in attP40), Δ*Tk^1^*, *10XUAS-Tk* were previously described in (Asahina et al., 2014). *20XUAS*>*myr:TopHAT2*>*CsChrimson:tdTomato* (in VK00022 and VK00005; Watanabe et al., 2017; Duistermars et al., 2018), *13XLexAop2*>*myr:TopHAT2*>*CsChrimson:tdTomato* (in attP2), *13XLexAop2-IVS-Syn21-GCaMP6f* (codon-optimized)*-p10* [in su(Hw) attP5 and su(Hw)attP1], and *13XLexAop2-IVS-syn21-shibire^ts^-p10* (in VK0005; Pfeiffer et al., 2012) were created by Barret Pfeiffer and provided by David Anderson (California Institute of Technology) and Gerald Rubin [Howard Hughes Medical Institute (HHMI) Janelia Research Campus]. *fru^FLP^* (RRID:BDSC_66870; Yu et al., 2010) was a gift from Barry Dickson (HHMI Janelia Research Campus). *pJFRC118-10XUAS-TLN:mCherry* (DenMark; in attP40) and *pJFRC67-3XUAS-IVS-Syt:GFP* [in Su(Hw)attP1; Seelig and Jayaraman, 2013] were a gift from David Anderson (California Institute of Technology). *trans*-Tango (in attP40; RRID:BDSC_77123; Talay et al., 2017) and *QUAS-mCD8:GFP* (Potter et al., 2010) were gifts from Mustafa Talay and Gilad Barnea (Brown University). *Tubulin-FRT-GAL80-FRT-stop* (Gordon and Scott, 2009) was a gift from Kristin Scott (University of California, Berkeley). *h-Cre* (Siegal and Hartl, 1996; RRID:BDSC_851), *vasa-Cas9* (Gratz et al., 2014; RRID:BDSC_51323), *VGlut-LexA:QFAD.2* (RRID:BDSC_60314), *ChAT-LexA:QFAD.0* (RRID:BDSC_60319), and *Gad1-LexA:QFAD.2* (RRID:BDSC_60324; Diao et al., 2015) flies were obtained from Bloomington Drosophila Stock Center (BDSC) at the University of Indiana.

### Creation of knock-in strains

*Takr86C^LexA^* and *Takr99D^LexA^* knock-in alleles were created using CRISPR/Cas9-mediated genome editing (Gratz et al., 2014). For both *TkR86C* and *TkR99D*, we first identified a pair of 21-nucleotide guide RNA (gRNA) sequences, using flyCRISPR Target Finder (http://targetfinder.flycrispr.neuro.brown.edu/) that are expected to delete the segment between the start codon and 3′ end of the first coding sequence-containing exon. The gRNA sequences are the following (PAM sequences are in the upper case): *TkR86C* gRNA #1, gcagtctgtaatcaggatag AGG; *TkR86C* gRNA #2, gtacttcctgcccactcact TGG; *TkR99D* gRNA 1, gaagtcactgcgattctcca TGG; and *TkR99D* gRNA #2, gtcataattaggcatgccgg CGG.

Two gRNA sequences for each gene were incorporated into the tandem gRNA expression vector pCFD4 following the protocol described in (Port et al., 2014). We call this plasmid a gRNA plasmid. In parallel, we also created a donor plasmid for each gene, using pHD-DsRed (catalog #51434, Addgene; Gratz et al., 2014) as a backbone. The donor plasmid contains the coding sequence of LexA:p65 (Pfeiffer et al., 2010) in frame with the start codon of *TkR86C* or *TkR99D*. These coding sequences are sandwiched by the 5′ UTR and the sequence immediately downstream of the start codon of *TkR86C* or *TkR99D*. The floxed 1,225 bp 3XP3-DsRed-SV40 marker gene was inserted in the orientation opposite to the targeted gene in the intron region of the 3′ arm (1294–70 bp downstream of the 3′ end of first exon of *TkR86C*, and 1293–69 bp downstream of the 3′ end of second exon of *TkR99D*). The start codon of *TkR86C* or *TkR99D* in the donor plasmid was changed to the amber stop codon (TAG). Also, the PAM motifs of the gRNA sequences on both arms within the donor plasmid were mutated to avoid secondary cleavage by Cas9 proteins. DNA fragments for both 5′- and 3′-homologous arms were amplified using PrimeSTAR GXL DNA polymerase (catalog #R050, Takara Bio) from the genome DNA of Canton-S wild-type strain of *Drosophila melanogaster*, which contained several point mutations and small indels compared with the standard *Drosophila* genome sequence. The 5′ arm and LexA:p65 coding sequence were assembled from two fragments from PCR-amplified *Drosophila* genome and a LexA:p65 coding sequence using NEBuilder HiFi DNA Assembly Cloning Kit (catalog #E5520, New England Biolabs), and inserted into XhoI-SpeI sites of the pHD-DsRed plasmid. The 3′ arm was subsequently inserted into NdeI-EcoRI sites of the intermediate plasmid using the same kit. The sequence of the plasmids that is expected to be incorporated into the fly genome (see Tables 2 and 3 for the full sequence) was verified by Sanger sequencing.

**Table 2.** DNA sequence of *TkR86C^LexA^* knock-in construct (pHD-DsRed-TkR86C^LexA^)

*TkR86C^LexA^* knock-in construct (pHD-DsRed-TkR86C^L^*^ex^*^A^)
The knock-in donor plasmid has the fragment below (including the floxed 3×P3-DsRed element) inserted between XhoI and EcoRI sites of the plasmid pHD-DsRed (catalog #51434, Addgene). XhoI and EcoRI sites are shown at the 5′ and 3′ end of the sequence, respectively. Point mutations that alter the start codon of *TkR86C* and PAM sequences are indicated in bold letters.
CTCGAG[XhoI]-CACAAACATTGGCAGACATTAGCAAAACACAAAATACGACAAACAAACGTACACAATCACAATGGGTCATTGGAACGGTCCTTGAATTGTTCATTGTTCGGGCAAGTGTGTGGGTGTTCCGCAGTAGGGTAATGTGTAAGTGGGGTGTGAACCGCGTCCTCTGCCATCATTTACAGGTCATTCATCACAGCACCACGAAGTGAAACCACACTTACACCCTCTCACCAAGTTTATGTAGTTGGGGTATCAGGTGTGCTGAACAAGGGGTTTTAAGCGGATTTTGGAGTTTCTACAATGAAAATAACAACTTGGTAAACCTTCGATAATTTACTTTTGGCCAGCACTCAAGAAAGTTTAGGAAGCCTTAGAAAACATCATATTTGCTTACAAGAGTTTTAAGCGTTCTCGTAAAGTTGCCATTATAAGGGAGAGTTTATTTTACTAGACGAATAGTTAATAACTTGAAAATTATTAAATTTACTTTTTAAAACGAAATTACCAATTTTTATTGTACAAAACATTCATTGAACCTATTTATTACTTGCTATAAAAACATATCCTGCAACATGTTGCCAGCGAAGTAATGTTGCTAGATTCTCTCAACAGGTATGTTTCCAGCAGAGGAACACTGCAGAATTGGTATTTTCATGCAAAGCATTCAGTCGGCGGCGGCACTTGCAGCGCTAAGGACACGCGTAGCACAGCGAACGGAAACTCGGGAGGCATTTTAAGAAAATAAGATTGTTGCCAACTAACGGTGTTTTCGTAGTGTGTGCGTGTGGGCTTGTTGCCACTGTATGAGTGTTGTGCTGGCTTAAAGCCAGCGAAAAGCGCAATGAAAAATAGTGGCCAAAAAAAGTGGTTACAAGGTGTGCAAAAAGTTTGTAATGTAAATACAAGTGGGTTGCAAAATATGTGCAGTGTTGTGATTTAAGAAATTCAATTTAAACGCTATTTATAGCATCAAATTGTGCGATAACTTT**G**GTAACATGTGATTAAAAGCATATTTTTTGTTCTAACGGAGTATAAATAGAAATGTTTTCTAAAAAAAAGGGGCTTCATATAAAAACAAAATGCAGATTAAGGTTTTATTAGTTCGTAATCTTCTGGATAAGTGTGAATAATAATTGGATACAATTAAACGAAGTATTGAAGTGAAACAATATACTTAAATGCAACCCATTTATGGAATCAAATTATTCCTATTTGATTTTCGAAGCAAGTTTAAATATTTAACTATATAAAATCCATGTGCGTTTAAACCTCAGATAATT**G**ACTTCGACACTTAAACTTATATTTAACGATTGCAAGGTTGACAGTTTTGTGACCAAAGGGCCACATGCAGTGTTAAACGCAACTCGTCCTTGACCACAGGCCAAAATTAAAAGTGTGTGTGCATGTGGCTGACAGTTCAAAGAACATAAAAAGGAAAGCTCCTTGGAAAACGCGCCACAAAAGAGGATCAACTGAAAGCACAGCATTTACGAAGAAATTAAGAGTCACCTTCCGG**G**AGAAATCCGTCGAGCTCAATGGTTTCTATGGC**G**CC**C**CGTTATTAAATGTGGCATAAGTGCAGCGGGATCATTACGGAAA**G**CAC**G**CTCTATCCTGATTACAGACTGCAAAATGCCACCCAAGAAGAAGCGAAAAGTAGAAGATCCAATGAAGGCTCTCACGGCCCGACAACAGGAAGTTTTTGATTTGATACGGGATCATATATCCCAAACGGGTATGCCTCCGACCCGCGCAGAGATAGCACAGCGACTGGGCTTTCGATCGCCTAACGCCGCGGAGGAGCACTTGAAGGCACTGGCCCGCAAGGGTGTCATTGAAATCGTGTCCGGTGCGAGCCGCGGAATCCGGCTGTTGCAGGAGGAGGAAGAGGGCCTGCCACTGGTGGGACGCGTGGCCGCTGGCGAGCCGCTGCTGGCCCAGCAACACATAGAGGGACACTATCAGGTGGACCCCTCCTTGTTTAAGCCAAATGCTGATTTCCTGTTGCGGGTGTCGGGAATGTCCATGAAGGACATCGGTATTATGGATGGTGACCTCCTCGCCGTCCATAAGACACAGGATGTCAGGAACGGCCAGGTAGTCGTTGCCAGGATAGACGATGAGGTCACTGTGAAACGTCTCAAGAAGCAAGGCAATAAGGTCGAGCTGCTGCCGGAGAATAGCGAGTTCAAGCCGATCGTGGTGGATCTGCGACAGCAGTCCTTTACTATCGAGGGCTTGGCCGTGGGTGTGATCCGCAACGGAGATTGGCTGGGATCCACGCCGATGGAGTTCCAGTACCTGCCCGATACGGATGACCGTCACCGTATCGAAGAAAAGCGGAAGCGAACCTATGAAACCTTCAAGTCCATCATGAAAAAGTCCCCCTTCTCGGGCCCCACGGACCCGCGCCCCCCGCCCCGTCGTATTGCGGTTCCTTCGCGCAGCAGTGCCAGCGTCCCCAAACCCGCACCGCAGCCCTACCCGTTCACTTCCTCCCTTAGCACGATTAACTATGATGAGTTCCCCACGATGGTGTTCCCCAGTGGACAAATTTCCCAGGCATCGGCACTGGCTCCGGCCCCACCGCAAGTCCTCCCCCAGGCGCCCGCTCCGGCACCGGCTCCCGCAATGGTGAGTGCTCTGGCCCAGGCCCCCGCTCCAGTCCCCGTGCTGGCGCCTGGACCCCCACAGGCAGTTGCCCCTCCTGCTCCGAAACCAACGCAGGCGGGCGAAGGAACCCTGAGCGAGGCCCTCTTGCAGCTTCAGTTCGATGACGAAGACTTGGGAGCCCTGCTGGGTAACAGCACAGACCCTGCCGTATTCACCGATCTCGCATCCGTGGACAACAGCGAGTTTCAGCAGCTCTTGAATCAGGGAATCCCGGTCGCACCTCATACCACAGAGCCCATGCTGATGGAATACCCGGAGGCTATCACGCGACTGGTGACCGGCGCACAGCGACCACCCGATCCAGCCCCTGCCCCACTGGGTGCCCCGGGTTTGCCCAATGGCCTCCTCAGCGGCGATGAGGATTTCTCCAGCATCGCTGATATGGATTTCTCCGCTTTGCTGAGCCAGATAAGCTCCTAA**TA**GTCGGAGATTGTCGACACCGAGCTGCTGGTCAACTGCACCATCCTCGCCGTCCGTCGATTCGAGCTGAATAGCATTGTGAACACCACGCTCCTGGGCAGTCTCAACAGAACCGAGGTGGTCAGCCTCTTGTCGAGCATTATCGACAATCGGGATAATCTCGAGAGCATCAATGAGGCCAAGTGAGT**C**GGCAGGAAGTACTGCGTCCTTTTATTTAGCCTTCCA**C**GTACTTCGACTACTAGTGCTCTTCTATAACTTCGTATAGCATACATTATACGAAGTTATACCGGTTAAGATACATTGATGAGTTTGGACAAACCACAACTAGAATGCAGTGAAAAAAATGCTTTATTTGTGAAATTTGTGATGCTATTGCTTTATTTGTAACCATTATAAGCTGCAATAAACAAGTTAACAACAACAATTGCATTCATTTTATGTTTCAGGTTCAGGGGGAGGTGTGGGAGGTTTTTTAAAGCAAGTAAAACCTCTACAAATGTGGTATGGCTGATTATGATCTAGAGTCGCGGCCCCTACAGGAACAGGTGGTGGCGGCCCTCGGCGCGCTCGTACTGCTCCACGATGGTGTAGTCCTCGTTGTGGGAGGTGATGTCCAGCTTGGAGTCCACGTAGTAGTAGCCGGGCAGCTGCACGGGCTTCTTGGCCATGTAGATGGACTTGAACTCCACCAGGTAGTGGCCGCCGTCCTTCAGCTTCAGGGCCTTGTGGATCTCGCCCTTCAGCACGCCGTCGCGGGGGTACAGGCGCTCGGTGGACGCCTCCCAGCCCATAGTCTTCTTCTGCATTACGGGGCCGTCGGAGGGGAAGTTCACGCCGATGAACTTCACCTTGTAGATGAAGGAGCCGTCCTGGAGGGAGGAGTCCTGGGTCACGGTCACCACGCCGCCGTCCTCGAAGTTCATCACGCGCTCCCACTTGAAGCCCTCGGGGAAGGACAGCTTCTTGTAGTCGGGGATGTCGGCGGGGTGCTTCACGTACACCTTGGAGCCGTACTGGAACTGGGGGGACAGGATGTCCCAGGCGAAGGGCAGGGGGCCGCCCTTGGTCACCTTCAGCTTGGCGGTCTGGGTGCCCTCGTAGGGGCGGCCCTCGCCCTCGCCCTCGATCTCGAACTCGTGGCCGTTCACGGAGCCCTCCATGCGCACCTTGAAGCGCATGAACTCCTTGATGACGTCCTCGGAGGAGGCCATGGTGGCGACCGGCTTCGAGCCGATTGTTTAGCTTGTTCAGCTGCGCTTGTTTATTTGCTTAGCTTTCGCTTAGCGACGTGTTCACTTTGCTTGTTTGAATTGAATTGTCGCTCCGTAGACGAAGCGCCTCTATTTATACTCCGGCGGTCGAGGGTTCGAAATCGATAAGCTTGGATCCTAATTGAATTAGCTCTAATTGAATTAGTCTCTAATTGAATTAGATCCCGTACGATAACTTCGTATAGCATACATTATACGAAGTTATGATCGCAGGTGTGCATATGTTCCACCGAGATCTTCTAGCAGCCCCCGCACAAAGTTATGCAAATTAAACGGTAGAGTTATCAGCGATTCGCGGAAGCGGCCAGTTTTCCCTGATGAAGGCTTAAATTGAATAAAGTTTCGTGGGTCTGGCAGCTGATAAGCACATTTGGCGTTCGTCCGGTTAAGTCGATGCACTTGGACTAAAAAGTAGAGTTACTTATACAAATTAGGATACGCAGCTATTGGATTTTCCTGCAAACTTTAATTGTTGATCAGCATTTTTTTGAGTGTTGTTTTGCAAGAGCAAACATTCTTAGTCTAAAATCTTGAAAGCCCTTTGGTAGCAGTAAGAAAATTGTGTACATTTGTTTAGGCCTAATCT**A**ATTGCTTTT**A**AAAGTAGATCCACAGCTCTTGGTCACGCTTAAATCATAAATGTTTTACGTACGCGGATTTCTTAAATACCGAAAACAAATCGTGGTATTCACACTCAATGTTCTTTAGTTGTCTTTCAACTATAATTTACAT**A**CGCTGCATGTTTCGTAAATTAATGCAATTTAAAAAAGGGTTTCATTGTGTAATTAAATTGTATCCTGTAAATTGCATATCCTTTATTAAAAAGCGAGAACAAATGAATGCTTCAAATAATAACAATTTATTCTGAGTGTTTTTGTTTTTTTAATTTTGCATATAGTTTTTAAAGCGATGTGGTGATTAGGGTATGACAGTTTAATTAAAAACAGATTCCACAAATAAGCGCAATTTCAATAAAATTATGATAATAGGATTAGTTAGACAAACACTTACATTGTTGAAATATCACACTTTGGATAAATACCTTCAGCATATACTCAGTTCAAATGAACGAGAAATTGGGATCCGTGGTAATTGAATTGATGATTAGCCTCTGAAGGAGTTGGCAAAGACAAATTTAATGCGTTGGTATGTTTTAATCAG**G**GCAATTATTTTAAGTT**G**ACCAAG**A**AAACCG**C**GTGTACTATAAATACCAGCGCTTTCATTTG**G**ATCGAAAGCTAACCGCAAAGTTAAATTAATGACTTGCCGCAATTGCGACTATTGCGTAATAACTCAAAAGTTTGCGCCATAACAAAATAAAAAAAA**A**CAAGGGCAATCGTTGAAAGTTCCAACCGGCGGATAAATATTCCCTCGACTTTGCCTTAATTATACCAGTGATGGATTTCGGTCTCTTTTGGAACTGGTTAATTAGGC**T**CTTAAACTGTTTTTCAGCCTCATTACAGGGGCTTTTGTTTTTGCTGGATATCCTTTAATTGTTTTGTAGCGGAAAATTCAAGAGGAGATTAAGTTATTTATGCTTTTATTTCGCTTTATTGGCCCACAACAGTCCGTACAAACTGGTTTATATATAAAATATATATAAATATAAAATATATAA**A**ATATATTATATATGACATGTTTGATAAAGAATATGTTATTGCTTATTATTTAAATTACAGGAAAATATGTATCGTTTTGCCTTA-[EcoRI]GAATTC

**Table 3.** DNA sequence of *TkR99D^LexA^* knock-in construct (pHD-DsRed-TkR99D^LexA^)

*TkR99D^LexA^* knock-in construct (pHD-DsRed-TkR99D^L^*^ex^*^A^)
The knock-in donor plasmid has the fragment below (including the floxed 3×P3-DsRed element) inserted between XhoI and EcoRI sites of the plasmid pHD-DsRed (catalog #51434, Addgene). XhoI and EcoRI sites are shown at the 5′ and 3′ end of the sequence, respectively. Point mutations that alter the start codon of *TkR99D* and PAM sequences are indicated in bold letters.
CTCGAG[XhoI]-TCCTGGGGCATTGACTGGGCTGATAGAGTGTTCTCCCG**G**CACACTAAACGGTGCACGGGGAG**A**AAAAAAT**T**ACTT**T**AAAC**T**TGATATCTCTTCTTTTAATATCTGATGAAACTAAATATCACTGAAAACACTTGCATTAAGGTGTC**G**AAAAGCATGCAACATAAAATGTGGTGCAAAT**A**CGCACGGCTTTAAAATTGTAGCATACTTTTGGGCTGCGCACCCAAGCCGCATGGATCCCTTTCACTTTCTGCGGTCGCCGGAATTTGGATGCGCTATTCCTGGCTTACAAATATTTTTCTGCCCATACCTTTAACTTTTTGCGAACAAACACAGTTGGAAATTTTTCCATTCCTGCTGGCTGGACAGTAGCAAAGTACGAGTATGAAAGCCAGCAAAGTAATAAAAACGCCTCCAAGGAGAGGAAAAGTTGCTACTCGGATTATCGTTTTGTCAGACTTCAAGTGACGCGACTGGCCGTCGCAATTATCTCGAGCTGCCAAGGACACATCCGAGGCATAATTGAAGGACGACCAGGTCGCTGGCGAGTAAGGACCTTTCCCCACTCCCCACTGCCCTTTCAAAACGAGAAAAAAGCCATCGAAGAGAGGGTTTACTGTCGTTCTGAAACAATTGTTTGCGAGCC**A**TCAGACACTCGAAAGGCAGGTGAACTCAGCTCTGGGGACTCATAAAAAGAAATAGCTCTGCTCGGCTTTTTCAATTGGTTTTGAGCTATTTTGGTCACATAATAACGCACAAGATACAG**T**TAATAA**A**AAATTTATTTAGGCTTTAGGGTCATTCGTCA**T**GTTAAATGGTTAATATCATTTAGCTAATAGAAAATTGTTTTTCATATAGCTTGAATAATATTTCACCAAGGAAATGTTTTTA**A**TAAAGCAGCTTTAGTTCTTTTCCGATTGCCATTCATTAGCGTTTACAGTCATAAATTAGTATTATTGGCCCGAATGCGAAAGTAAAAGTTCGCACTGGACAAATCACCG**A**CATTTAGCGCTCCC**A**AAGTTTCCAAGGTATCCGATCCACTAATGGCCACCACATCGCAACCTCATCAAGAGGCACATACTTGGCAACTCTGCCGGGGGACGAGTAGTT**CT**AGGTTCTAGATTATTCAAGGCACTAGAAGGAAACCATCGAAAAGTTCTTGGGAGCAGGAGCAG**G**AGATAGGGCGAAAATGAGTCACGTAG**A**GCGAGAAGAGAAACAAACTCAAGAAGAAATCAAAATCGATATGAATCCCTTCATGAGCACGTAGCAGGAATGTGCAAAGTTTTCACTGAAAATG**C**GCATCAAAAAGCATTGTTTGCAACAAACAGC**C**ACCAAGAGCAGCCAAACACGAGGCCATCAAAGATCAC**CA**AAACCAAAAAAAA**AA**GAAATAAAATAAAACGAAACGAAACATGCCAGAAATGAAATTAGA**A**GGTC**T**TGCCA**A**CGTTTTCTTTGCATTTTTCAGGTCGAGCAGTTGGCAAATGGAAAGCAAATAAGGCAAGGCAAAGGGATACGCAACATTAAGTCCGGAATCGTTCAGTCCGGATAAAGACAAAGGAAAACACTCCGGGGTGCGGAAAACAACATCAGCAGCAGCAGCACCAGCAGCAGCCATGCCACCCAAGAAGAAGCGAAAAGTAGAAGATCCAATGAAGGCTCTCACGGCCCGACAACAGGAAGTTTTTGATTTGATACGGGATCATATATCCCAAACGGGTATGCCTCCGACCCGCGCAGAGATAGCACAGCGACTGGGCTTTCGATCGCCTAACGCCGCGGAGGAGCACTTGAAGGCACTGGCCCGCAAGGGTGTCATTGAAATCGTGTCCGGTGCGAGCCGCGGAATCCGGCTGTTGCAGGAGGAGGAAGAGGGCCTGCCACTGGTGGGACGCGTGGCCGCTGGCGAGCCGCTGCTGGCCCAGCAACACATAGAGGGACACTATCAGGTGGACCCCTCCTTGTTTAAGCCAAATGCTGATTTCCTGTGGCTGTTGCAGGAGGAGGAAGAGGGCCTGCCACTGGTGGGACGCGTGGCCGCTGGCGAGCCGCTGCTGGCCCAGCAACACATAGAGGGACACTATCAGGTGGACCCCTCCTTGTTTAAGCCAAATGCTGATTTCCTGTTGCGGGTGTCGGGAATGTCCATGAAGGACATCGGTATTATGGATGGTGACCTCCTCGCCGTCCATAAGACACAGGATGTCAGGAACGGCCAGGTAGTCGTTGCCAGGATAGACGATGAGGTCACTGTGAAACGTCTCAAGAAGCAAGGCAATAAGGTCGAGCTGCTGCCGGAGAATAGCGAGTTCAAGCCGATCGTGGTGGATCTGCGACAGCAGTCCTTTACTATCGAGGGCTTGGCCGTGGGTGTGATCCGCAACGGAGATTGGCTGGGATCCACGCCGATGGAGTTCCAGTACCTGCCCGATACGGATGACCGTCACCGTATCGAAGAAAAGCGGAAGCGAACCTATGAAACCTTCAAGTCCATCATGAAAAAGTCCCCCTTCTCGGGCCCCACGGACCCGCGCCCCCCGCCCCGTCGTATTGCGGTTCCTTCGCGCAGCAGTGCCAGCGTCCCCAAACCCGCACCGCAGCCCTACCCGTTCACTTCCTCCCTTAGCACGATTAACTATGATGAGTTCCCCACGATGGTGTTCCCCAGTGGACAAATTTCCCAGGCATCGGCACTGGCTCCGGCCCCACCGCAAGTCCTCCCCCAGGCGCCCGCTCCGGCACCGGCTCCCGCAATGGTGAGTGCTCTGGCCCAGGCCCCCGCTCCAGTCCCCGTGCTGGCGCCTGGACCCCCACAGGCAGTTGCCCCTCCTGCTCCGAAACCAACGCAGGCGGGCGAAGGAACCCTGAGCGAGGCCCTCTTGCAGCTTCAGTTCGATGACGAAGACTTGGGAGCCCTGCTGGGTAACAGCACAGACCCTGCCGTATTCACCGATCTCGCATCCGTGGACAACAGCGAGTTTCAGCAGCTCTTGAATCAGGGAATCCCGGTCGCACCTCATACCACAGAGCCCATGCTGATGGAATACCCGGAGGCTATCACGCGACTGGTGACCGGCGCACAGCGACCACCCGATCCAGCCCCTGCCCCACTGGGTGCCCCGGGTTTGCCCAATGGCCTCCTCAGCGGCGATGAGGATTTCTCCAGCATCGCTGATATGGATTTCTCCGCTTTGCTGAGCCAGATAAGCTCCTAA**TA**GGAGAATCGCAGTGACTTCGAGG CGGATGACTACGGCGGAGCAATTGGAGCAACTGGAGCACCCCCGCCGGCGTCCTTTTCTCGGCCATGAGCAGCGTGCTCTCGGCCAGCAACCATACGCCTCTGCCGGACTTTGGCCAGGAGCTCGCCCTATCCACCAGCTCCTTCAATCACAGCCAGACGTGAGTTGAACTCGGATC**G**GCCGGCATGCCTAATTATGACACAAACTCAATTAAACTAGTGCTCTTCTATAACTTCGTATAGCATACATTATACGAAGTTATACCGGTTAAGATACATTGATGAGTTTGGACAAACCACAACTAGAATGCAGTGAAAAAAATGCTTTATTTGTGAAATTTGTGATGCTATTGCTTTATTTGTAACCATTATAAGCTGCAATAAACAAGTTAACAACAACAATTGCATTCATTTTATGTTTCAGGTTCAGGGGGAGGTGTGGGAGGTTTTTTAAAGCAAGTAAAACCTCTACAAATGTGGTATGGCTGATTATGATCTAGAGTCGCGGCCCCTACAGGAACAGGTGGTGGCGGCCCTCGGCGCGCTCGTACTGCTCCACGATGGTGTAGTCCTCGTTGTGGGAGGTGATGTCCAGCTTGGAGTCCACGTAGTAGTAGCCGGGCAGCTGCACGGGCTTCTTGGCCATGTAGATGGACTTGAACTCCACCAGGTAGTGGCCGCCGTCCTTCAGCTTCAGGGCCTTGTGGATCTCGCCCTTCAGCACGCCGTCGCGGGGGTACAGGCGCTCGGTGGACGCCTCCCAGCCCATAGTCTTCTTCTGCATTACGGGGCCGTCGGAGGGGAAGTTCACGCCGATGAACTTCACCTTGTAGATGAAGGAGCCGTCCTGGAGGGAGGAGTCCTGGGTCACGGTCACCACGCCGCCGTCCTCGAAGTTCATCACGCGCTCCCACTTGAAGCCCTCGGGGAAGGACAGCTTCTTGTAGTCGGGGATGTCGGCGGGGTGCTTCACGTACACCTTGGAGCCGTACTGGAACTGGGGGGACAGGATGTCCCAGGCGAAGGGCAGGGGGCCGCCCTTGGTCACCTTCAGCTTGGCGGTCTGGGTGCCCTCGTAGGGGCGGCCCTCGCCCTCGCCCTCGATCTCGAACTCGTGGCCGTTCACGGAGCCCTCCATGCGCACCTTGAAGCGCATGAACTCCTTGATGACGTCCTCGGAGGAGGCCATGGTGGCGACCGGCTTCGAGCCGATTGTTTAGCTTGTTCAGCTGCGCTTGTTTATTTGCTTAGCTTTCGCTTAGCGACGTGTTCACTTTGCTTGTTTGAATTGAATTGTCGCTCCGTAGACGAAGCGCCTCTATTTATACTCCGGCGGTCGAGGGTTCGAAATCGATAAGCTTGGATCCTAATTGAATTAGCTCTAATTGAATTAGTCTCTAATTGAATTAGATCCCGTACGATAACTTCGTATAGCATACATTATACGAAGTTATGATCGCAGGTGTGCATATGTAATCAAACTCACGCACGAACAGAGCACTCGAATCATGGGAGTCTGGAACGTTCGGTTCAGGGAGGCTATCCATTTCTAGACCACCTTTAATATTTGTTTATTATTTTATATATATTTTCATTTCCCTTTCTCCCCTTCAAACCAATGAAACTTCTCATTTAAAGCGATATCAATTAAAAGAGTCGTAAAATACGCCTGCTACTTCATTGCTCCCAAATGGGGGATCCCTTACATTGCGTGGCTTTTTGATGGGTCCTGATGAGTCCGCCTCCGTTTCGGTTTTATCCGCGATTCAGCCATGATTGTGCCCACACCCACGTGCATCTCTAATGACGCCTGCGTTCAGTCATTCCGGCATTCAGTCATCCACTGGGGCATTTCCCTTCCGCTCATCACTTCCGTTTGAAGTACACAAAACGGCGGAGAAGGCAAACACATTTTATGCGGAGACACTTACTTTTTCCGCATACATATATACACAAAAAACTGCGGGTCTATGGCATTCAACTCTATTCTTTCCGGCTGAAAAAACGTCGGCACTTTTTGCAGACATGTCCCAAATTTACTGCCCCACCAGCACCAACAAATGGGTTTCGCTGGCACTGGAAGTGGAATAATTATTTGGATATGTGGCTATGTGGATATGGGAATATGGCTGCGTCTGACGCATCGAAAATCCATTCTGCGAATAACTTTGGTTTTTTTTGCTGGCCAAACGGAGGCCACATATATCTGCCTTCAGGCAGTGCTCAACAGTCGATCCTGGTAGTTAACTGGCTGCTCTTTTTCCCATCCGAGTTGTCCATTTCTGATGTCCGATTTATGTGCAAATAGAGACGGCCAAGATGGCTTAAAATTGGCAGGACGAAGGACTCAGCCTGGCATATTTTTCGGCACAAAAGAATCCGTGCATCAAAGCGTAAACATATTTGTACACATTCCTCTGACTGAAGTGGCACAATCACAATTTATTTGCCAAACTTTCCACATTCCCATAACAACTAATATGGAATACATGGGCTAATTGCTTTGATGGTCTATTTCTGGCTGAGGTCACACGTATTTGTTTGAAAAGAAGAATATATTTTTAAGGGAGAAAGTATTTACAACAATGGAACAATAGGTCTTCACTGTGGCATAAAAAGAACTTTTTAAATTGACTTTTTCCATCGTTATCAGGCATTTTAGAGGTATTTATTCTATCTTAAAGATCTTGCTTGCAATATTATTTATTAATTTTAGGAAACTTCCCACACCGACTCACATTTCTCTGTACTATTTAAACCGCAGCAGCAATATCAACAAGAATATAAGCTATGGCATAGTGAAAATATTTTTGTCGTGACATTTTCTTTGGCCCAAGGCATTCTGTTTTCCCATTTTCCTCGGCAGCCATTTCCCATTCAGCTCGTCATAGGCCTGTCGAGGCCCCACAAAAAACAAATCAATAATCCTCTTTGCATTTGGGAGCTCGAACAAAGGGCATTTTATGTAAATCCCCGCAGTTTGTCATGCGAAAAGGGTGAATGGGGTGTGTAA-[EcoRI]GAATTC

The appropriate combination of gRNA and donor plasmids was mixed and injected into embryos of *vasa-Cas9* strain (stock #51323, BDSC) by BestGene. G1 adults (offspring of injected G0 animals) were screened for the presence of DsRed expression in the compound eyes, followed by PCR screening. The Southern blotting was used to verify the correct integration of the donor element (see below). After backcrossing the knock-in alleles in a Canton-S background for six generations, the 3XP3-DsRed marker gene was removed by using *Cre* recombinase. Specifically, flies containing the knock-in allele crossed to flies that express the *hs-Cre* transgene (Siegal and Hartl, 1996). This transgene induced efficient excision of the floxed marker gene under the standard rearing temperature of 25°C, as *hs-Cre* was previously reported to be active without heat shock (Siegal and Hartl, 1996; Hampel et al., 2011). The offspring were screened for the loss of DsRed expression in the eyes.

### Creation of transgenic strains

The *15XQUAS-GCaMP6f* [in su(Hw)attP5 and su(Hw)attP1] transgenic strains were created in the following steps. First, a DNA fragment that contains IVS-Syn21-GCaMP6f (codon optimized)-p10 elements was amplified from the genomic DNA of the transgenic strain that carries *13XLexAop2-IVS-Syn21-GCaMP6f-p10* [in su(Hw)attP1] by PCR (Phusion Green, catalog #F534, Thermo Fisher Scientific). This fragment was subcloned into pCR Blunt II TOPO vector using the Zero Blunt TOPO kit (catalog #K287540, Thermo Fisher Scientific). In parallel, a modified version of the plasmid pJFRC164-21XUAS-KDRT>-dSTOP-KDRT>-myr::RFP (catalog #32141, Addgene), in which the 21XUAS element was replaced with a 13XLexAop2 element, was digested with XhoI and EcoRI. The IVS-Syn21-GCaMP6f-p10 element in the pCR Blunt II TOPO vector was amplified with overhang sequences and ligated into the digested backbone of the modified pJFRC164 using the In-Fusion HD Cloning Kit (catalog #639648, Takara Bio) to create the plasmid 13XLexAop2-KDRT>-dSTOP-KDRT>-IVS-Syn21-GCaMP6f-p10 intermediate plasmid (named pMW02). Next, the 15XQUAS sequence from the plasmid pBAC-ECFP15XQUAS-TATA-mCD8:GFP-SV40 (catalog #104878, Addgene) was amplified by PCR, which was subsequently used to replace the LexAop2 sequence of pMW02, which was excised by HindIII and AatII, using the In-Fusion HD Cloning Kit. The resulting plasmid, 15XQUAS- KDRT>-dSTOP-KDRT>-IVS-Syn21-GCaMP6f-p10, was then digested with AatII and NotI to remove the KDRT cassette, which was replaced by a Hsp70-IVS fragment excised by AatII and NotI from the plasmid pJFRC28-10XUAS-IVS-GFP-p10 (catalog #36431, Addgene). The sequence of the final product [15XQUAS-Hsp70-IVS-Syn21-GCaMP6f (codon-optimized)-p10, shorthanded as 15XQUAS-GCaMP6f; see Table 4 for the full sequence] was verified before being integrated into target attP sites via phiC31-mediated site-specific transformation (BestGene).

**Table 4.** DNA sequence of 15QUAS-Hsp70-IVS-Syn21-GCaMP6f (codon optimized)-p10 construct (pJFRC-15QUAS-IVS-OpGCaMP6f-p10)

15×QUAS-Hsp70-IVS-Syn21-GCaMP6f (codon optimized)-p10 construct (pJFRC-15×QUAS-IVS-OpGCaMP6f-p10)
The plasmid used for embryo injection has the fragment below inserted between HindIII and EcoRI sites of the plasmid pJFRC164-21×UAS-KDRT>-dSTOP-KDRT>-myr::RFP (catalog #32141, Addgene), which is the backbone common to all pJFRC reporter vectors (Pfeiffer et al., 2010). HindIII and EcoRI sites are shown at the 5′ and 3′ end of the sequence, respectively.
AAGCTT[HindIII]-ACTGGGTCAGTGGGTAATCGCTTATCCTCGGATAAACAATTATCCTCACGGGTAATCGCTTATCCGCTCGGGTAATCGCTTATCCTCGGGTAATCGCTTATCCTTGGGTAATCGCTTATCCTCGGATAAACAATTATCCTCACGGGTAATCGCTTATCCGCTCGGGTAATCGCTTATCCTCGGGTAATCGCTTATCCTTGGGTAATCGCTTATCCTCGGATAAACAATTATCCTCACGGGTAATCGCTTATCCGCTCGGGTAATCGCTTATCCTCGGGTAATCGCTTATCCTTTCACGTTGGGGACGTCGAGCGCCGGAGTATAAATAGAGGCGCTTCGTCTACGGAGCGACAATTCAATTCAAACAAGCAAAGTGAACACGTCGCTAAGCGAAAGCTAAGCAAATAAACAAGCGCAGCTGAACAAGCTAAACAATCTGCAGTAAAGTGCAAGTTAAAGTGAATCAATTAAAAGTAACCAGCAACCAAGTAAATCAACTGCAACTACTGAAATCTGCCAAGAAGTAATTATTGAATACAAGAAGAGAACTCTGAATAGATCTAAAAGGTAGGTTCAACCACTGATGCCTAGGCACACCGAAACGACTAACCCTAATTCTTATCCTTTACTTCAGGCGGCCGCGGCTCGAGAACTTAAAAAAAAAAATCAAAATGGGATCGCATCACCACCATCATCACGGAATGGCGTCCATGACAGGCGGACAACAGATGGGACGAGATTTGTACGATGATGACGACAAGGATCTGGCGACGATGGTGGATAGCTCGCGCAGGAAGTGGAACAAGACGGGTCACGCCGTTCGTGCCATAGGACGGCTCAGTTCGCTTGAGAATGTGTATATTAAGGCGGACAAACAGAAGAACGGTATAAAAGCCAATTTCAAGATACGTCATAACATTGAGGATGGCGGTGTGCAGCTCGCATATCACTACCAGCAGAACACTCCCATAGGTGATGGCCCGGTGCTCCTTCCCGACAACCACTATCTCTCCGTACAGTCGAAGTTGTCGAAAGATCCCAACGAGAAGCGGGACCACATGGTGCTGTTGGAGTTTGTTACGGCCGCGGGTATCACCCTGGGAATGGACGAGTTGTATAAGGGTGGAACTTAAAAAAAAAAATCAAAAACCGGCGGCAGTATGGTGAGTAAGGGCGAGGAGCTCTTCACCGGCGTAGTTCCGATACTCGTAGAGTTGGATGGCGACGTGAACGGCCACAAGTTCTCCGTCAGTGGAGAGGGCGAGGGCGATGCCACGTACGGAAAGTTGACGCTGAAATTTATCTGCACGACGGGCAAATTGCCGGTGCCATGGCCGACCCTCGTGACAACGTTGACTTACGGAGTGCAGTGCTTCTCGCGCTATCCAGACCACATGAAGCAACACGACTTTTTCAAGTCGGCCATGCCGGAGGGCTATATTCAAGAGCGTACGATATTTTTCAAGGATGATGGTAATTACAAGACCCGAGCCGAGGTCAAGTTTGAGGGAGATACACTGGTGAACCGAATAGAGCTCAAGGGCATCGATTTTAAGGAGGACGGCAATATCCTGGGCCATAAACTGGAATACAACCTCCCTGACCAGCTCACCGAGGAACAGATTGCCGAGTTCAAGGAAGAATTCTCGCTGTTCGATAAGGATGGTGACGGCACCATAACCACCAAGGAACTGGGTACGGTGATGCGCTCCTTGGGTCAAAACCCCACCGAGGCCGAGCTCCAGGACATGATCAACGAAGTAGACGCCGACGGCGACGGCACGATTGACTTCCCAGAGTTCCTTACAATGATGGCACGCAAAATGAAGTACCGCGACACAGAGGAGGAGATTCGAGAGGCCTTCGGAGTATTCGACAAGGACGGCAACGGTTACATCAGCGCCGCAGAGCTGCGGCATGTGATGACGAACCTGGGTGAAAAGCTGACCGATGAAGAAGTCGACGAAATGATCAGGGAGGCAGATATAGATGGTGATGGTCAGGTGAACTACGAGGAATTTGTTCAGATGATGACCGCTAAGTAATCTAGAATGAATCGTTTTTAAAATAACAAATCAATTGTTTTATAATATTCGTACGATTCTTTGATTATGTAATAAAATGTGATCATTAGGAAGATTACGAAAAATATAAAAAATATGAGTTCTGTGTGTATAACAAATGCTGTAAACGCCACAATTGTGTTTGTTGCAAATAAACCCATGATTATTTGATTAAAATTGTTGTTTTCTTTGTTCATAGACAATAGTGTGTTTTGCCTAAACGTGTACTGCATAAACTCCATGCGAGTGTATAGCGAGCTAGTGGCTAACGCTTGCCCCACCAAAGTAGATTCGTCAAAATCCTCAATTTCATCACCCTCCTCCAAGTTTAACATTTGGCCGTCGGAATTAACTTCTAAAGATGCCACATAATCTAATAAATGAAATAGAGATTCAAACGTGGCGTCATCGTCCGTTTCGACCATTTCCGAAAAGAACTCGGGCATAAACTCTATGATTTCTCTGGACGTGGTGTTGTCGAAACTCTCAAAGTACGCAGTCAGGAACGTGCGCGACATGTCGTCGGGAAACTCGCGCGGAAACATGTTGTTGTAACCGAACGGGTCCCATAGCGCCAAAACCAAATCTGCCAGCGTCAATAGAATGAGCACGATGCCGACAATGGAGCTGGCTTGGATAGCGATTC-[EcoRI]GAATTC

The *LexAop2-TkR86C* transgenic element was created by replacing the myr:GFP coding sequence of the plasmid pJFRC19-13XLexAop2-IVS-myr::GFP (catalog #26224, Addgene) with the coding sequence of *TkR86C*. Specifically, a DNA fragment of the *TkR86C* coding region was amplified from cDNA from the Canton-S wild-type strain by PCR (PrimeSTAR GXL, Takara Bio) with primers that had NotI and XbaI sites at 5′ and 3′ ends, respectively. The fragment was subcloned into the pCR Blunt II TOPO vector. pJFRC19 plasmids and *TkR86C*-containing vector plasmids were digested with NotI and XbaI. The pJFRC19 backbone and *TkR86C* fragments were ligated using Roche Rapid DNA Ligation Kit (catalog #11635379001, Millipore Sigma). The recovered *TkR86C* coding sequences (isoform B, 1,665 bp) have three base substitutions, including one nonsynonymous mutation (T425I), compared with the National Center for Biotechnology Information reference sequence NP_001097741.1. The HA-tagged version was created by adding the 135 bp that contains a 3× repeat of the hemagglutinin sequence at the C terminus of the *TkR86C* coding sequence. The coding region (see Tables 4 and 5 for full sequences) was fully sequenced before transformation.

**Table 5.** DNA sequence of 13LexAop2-IVS-TkR86C/TkR86C:HA constructs (pJFRC-13LexAop2-IVS-TkR86C/TkR86C:HA)

13×LexAop2-IVS-TkR86C/TkR86C:HA constructs (pJFRC-13×LexAop2-IVS-TkR86C/TkR86C:HA)
The plasmid used for embryo injection has the fragment below inserted between NotI and XbaI sites of the plasmid pJFRC19-13×LexAop2-IVS-myr::GFP (catalog #32141, Addgene), which is the backbone common to all pJFRC reporter vectors (Pfeiffer et al., 2010). NotI and XbaI sites are shown at the 5′ and 3′ end of the sequence, respectively. The HA tag sequence is present only in the 13XLexAop2-IVS-TkR86C:HA construct. The 3 point mutations are indicated in bold letters.
GCGGCCGC[NotI]-CAAAATGTCGGAGATTGTCGACACCGAGCTGCTGGTCAACTGCACCATCCTCGCCGTCCGTCGATTCGAGCTGAATAGCATTGTGAACACCACGCTCCTGGGCAGTCTCAACAGAACCGAGGTGGTCAGCCTCTTGTCGAGCATTATCGACAATCGGGATAATCTCGAGAGCATCAATGAGGCCAAAGACTTTCTGACCGAGTGCCTGTTTCCATCGCCGACAAGACCGTACGAGTTGCCATGGGAGCAGAAAACGATTTGGGC**C**ATAATTTTCGGTCTGATGATGTTTGTGGCCATTGCTGGCAATGGTATTGTTCTCTGGATCGTTACAGGACATCGCAGCATGAGGACGGTCACAAATTACTTCCTGCTGAACTTGAGCATCGCCGACCTGCTGATGTCGTCGCTGAACTGCGTCTTCAACTTTATATTCATGCTGAACTCAGATTGGCCATTCGGTTCGATTTATTGCACAATCAACAATTTCGTGGCCAACGTCACGGTCTCTACGTCGGTCTTCACGCTCGTGGCCATAAGTTTCGATAGATACATCGCCATTGTGCATCCGCTGAAACGCCGCACGTCGCGTCGGAAGGTGCGCATCATCCTGGTCCTGATCTGGGCACTCAGCTGTGTGCTGTCGGCGCCATGTCTGCTCTACTCCAGCATCATGACCAAGCACTATTACAATGGAAAATCGAGGACAGTCTGCTTCATGATGTGGCCAGATGGACGATATCCCACTTCTATGG**T**GGATTATGCATACAACCTGATCATCCTGGTACTGACCTACGGCATTCCCATGATTGTGATGCTCATATGCTACTCTCTCATGGGTCGTGTCCTGTGGGGCAGTAGATCAATCGGCGAGAACACGGATCGCCAGATGGAGTCGATGAAGTCGAAGCGGAAGGTGGTGCGCATGTTTATTGCCATCGTGTCCATCTTTGCCATTTGCTGGCTGCCGTATCACCTGTTCTTCATCTACGCCTACCACAACAACCAGGTGGCATCCACGAAGTACGTGCAACATATGTATCTCGGTTTCTACTGGCTGGCCATGTCCAATGCTATGGTCAATCCGCTCATTTACTACTGGATGAATAAGAGGTTCCGGATGTACTTCCAGCGGATCATCTGCTGCTGTTGCGTGGGCCTCACCCGCCATCGATTCGACTCGCCGAAGAGCCGGTTGACGAACAAGAACAGCTCGAACCGGCACACAAGAGGTGGGTACACCGTCGCCCACTCGCTCCCCAACTCCTCCCCCCCGACCA**T**CCAAACTCTTTTGGCCGTCCTGGCCCAGACTCTGACTCAGCCTAAGCCTCAGACCCAGTTGCTCTTGTCCCACCACTCACCACATCCCACGCAACCTTCCGCAGCGGAGACCAAGAGTCAGTGGAAGCGCAGCACGATGGAGACGCAGATCCAGCAGGCGCCGGTCACCAGCTCTTGCCGGGAGCAGCGGAGTGCACAGCAGCAGCAGCCACCCGGAAGTGGAACCAATCGGGCAGCCGTCGAGTGTATTATGGAACGGCCGGCGGATGGATCCAGTTCGC
CGCTGTGCCTTTCGATCAACAACAGCATCGGTGAGCGGCAGCGCGTGAAAATCAAATACATCTCCTGTGACGAGGACAATAATCCCGTGGAGCTCAGTCCCAAGCAGATG[HA tag sequence begins]ATGGATCTCCACCGCGGTGGAGGCCGCATCTTTTACCCATACGATGTTCCTGACTATGCGGGCTATCCCTATGACGTCCCGGACTATGCAGGATCCTATCCATATGACGTTCCAGATTACGCTGCTCATGGCGGATGAC[HA tag sequence ends]-[XbaI]TCAGA

### Southern blotting

Two hundred adult flies per genotype were homogenized in 800 µl of Tris-EDTA (TE) buffer (Tris/HCl, pH 9, 100 mm EDTA) supplemented with 1% SDS, followed by incubation at 65°C for 30 min. Three hundred µl of 3 m potassium acetate was added to the mixture, which was subsequently placed on ice for 30 min. After centrifugation at 13,000 rpm for 20 min at 4°C, the supernatant (∼600 µl) was collected and mixed with a half volume of isopropanol. Samples were centrifuged at 13,000 rpm for 10 min, and the pellet was washed with 70% ethanol. Precipitates were dried and dissolved in 500 µl of TE buffer. Samples were then treated with RNase A (0.4–0.8 mg/ml) at 37°C for 15 min. For purification, each sample was mixed vigorously with the same volume of phenol/chloroform/isoamyl alcohol (25:24:1 v/v; catalog #516726, Millipore Sigma).

After centrifugation at 13,000 rpm for 5 min, the aqueous upper layer was collected and mixed vigorously with the same volume of chloroform, followed by another centrifugation at 13,000 rpm for 5 min. The upper layer (∼400 µl) was further subjected to ethanol precipitation. The final precipitates obtained were dried and dissolved in 100 µl of TE buffer. The typical yield of genomic DNA extracted from 200 flies was 0.2–0.5 mg. Ten to 20 μg of genomic DNA per genotype was digested with a restriction enzyme (BglII for characterizing the *TkR86C^LexA^* allele, XhoI for characterizing the *TkR99D^LexA^* allele) at 37°C overnight. Electrophoresis was performed using a 0.7% agarose gel. Roche Digoxigenin (DIG)-labeled DNA Molecular Weight Marker III (catalog #11218603910, Millipore Sigma) was loaded as a marker. The gel, placed on a shaker within an empty pipette tip box, was sequentially subjected to depurination (in 0.25N HCl for 10 min), denaturation (in 0.5 m NaOH, 1.5 m NaCl for 15 min × 2), neutralization (in 0.5 m Tris/HCl, pH 7.5), 1.5 m NaCl for 15 min × 2), and equilibration (in 20× SSC for 10 min). DNA was transferred to a nylon membrane (catalog #11209299001, Millipore Sigma) overnight by sandwiching the gel and membrane between paper towels soaked in 20× SSC under a 1.5 k weight. DNA was immobilized onto the membrane by using a Stratalinker 2400 UV Crosslinker.

DIG-labeled DNA probes were synthesized using a Roche PCR DIG Probe Synthesis Kit (catalog #11636090910, Millipore Sigma). Probes were designed to target either the LexA coding sequence (Probe 1, 660–1280 bp downstream from the start codon of the nls:LexA:p65; see Table 6 for full sequence) or the flanking genomic region specific for each gene. For *TkR86C*, the probe (Probe 2; see Table 7 for full sequence) was targeted to the genomic region 2054–1733 bp upstream of the 5′ end of the exon 1. For *TkR99D*, the probe (Probe 3; see Table 8 for full sequence) was targeted to the genomic region 1814–2,317 bp downstream from the 3′ end of the exon 2. The DIG-labeled probes were hybridized to the membrane in Roche DIG Easy Hyb hybridization buffer (catalog #11603558001, Millipore Sigma) at 49°C overnight. The membrane was sequentially washed twice with a low stringency buffer (2× SSC, 0.1% SDS) at room temperature for 5 min, and then twice with a prewarmed high stringency buffer (5× SSC, 0.1% SDS) at 68°C for 15 min. After another brief wash with a DIG Easy Hyb kit wash buffer, the membrane was soaked in a DIG Easy Hyb blocking buffer at 4°C overnight. Roche anti-DIG-alkaline phosphatase Fab fragments (catalog #11093274910, Millipore Sigma) were added to the blocking buffer at 1:10,000, and the membrane was incubated at room temperature for 30 min. The membrane was washed with the wash buffer for 15 min, twice, followed by a brief equilibration in a DIG Easy Hyb kit detection buffer. As a chemiluminescence substrate, Roche CDP-Star (catalog #11759051001, Millipore Sigma) was freshly diluted to 1:200 in the same buffer. Signals were developed on autoradiography films (catalog #30-507, Genesee Scientific).

**Table 6.** DNA sequence of Southern blotting Probe 1 (621 bp, targeted to the LexA coding sequence)

Southern blotting Probe 1 (621 bp, targeted to the LexA coding sequence)
GTTCCAGTACCTGCCCGATACGGATGACCGTCACCGTATCGAAGAAAAGCGGAAGCGAACCTATGAAACCTTCAAGTCCATCATGAAAAAGTCCCCCTTCTCGGGCCCCACGGACCCGCGCCCCCCGCCCCGTCGTATTGCGGTTCCTTCGCGCAGCAGTGCCAGCGTCCCCAAACCCGCACCGCAGCCCTACCCGTTCACTTCCTCCCTTAGCACGATTAACTATGATGAGTTCCCCACGATGGTGTTCCCCAGTGGACAAATTTCCCAGGCATCGGCACTGGCTCCGGCCCCACCGCAAGTCCTCCCCCAGGCGCCCGCTCCGGCACCGGCTCCCGCAATGGTGAGTGCTCTGGCCCAGGCCCCCGCTCCAGTCCCCGTGCTGGCGCCTGGACCCCCACAGGCAGTTGCCCCTCCTGCTCCGAAACCAACGCAGGCGGGCGAAGGAACCCTGAGCGAGGCCCTCTTGCAGCTTCAGTTCGATGACGAAGACTTGGGAGCCCTGCTGGGTAACAGCACAGACCCTGCCGTATTCACCGATCTCGCATCCGTGGACAACAGCGAGTTTCAGCAGCTCTTCAGGGAATCCCGGTCGCACCTCATACCACAGAGCCCAT

**Table 7.** DNA sequence of Southern blotting Probe 2 (322 bp, targeted to TkR86C genomic region)

Southern blotting Probe 2 (322 bp, targeted to *TkR86C* genomic region)
ACACATGCCAGACGGTTCTGATTAATTTCTTGAAGCCTTTTGATTGGTGGGGAAAACATATATATCTGCCGGTAATTATGTTTTTGCGGTTACCAATTAATTTGCATATGCAATGCTGACTCGCAGTTCGCGCTTCGCTGGCTGCCAAAGGGATTTCCACCAGCACATGCCCTCTGGCGAAAACTTTTCCCCTGCATATTTTTCTAACCGCACCGCAACCATAAATCAGCACTAAAGTTTGTTGCCATCGACTGGCGACAGAATCAATGGGAATCTCCCCATGATGCACACACGGCGTATACTTGATTTCGCACCAAGAACC

**Table 8.** DNA sequence of Southern blotting Probe 3 (504 bp, targeted to TkR99D genomic region)

Southern blotting Probe 3 (504 bp, targeted to *TkR99D* genomic region)
CGAGATTGCCAACCCTCTATTTGAACACTCTCCTAGATGCGAGCCTAAGCAAACAAGCCAGGACCCACAAAATATTAAAAAGATGACGACAGCCAACGATGAGTGAGTGGAAAGGAATGGAGTGGATGGAGTTGAGTAGATGAGGAGTGGGACTCCTGCCCTCCGGCTTAGCTGAACCCCATGTTTTATGTGGCAGACGGCGCTGCAGAAAAACACGGATTTGTCAACGCACGCACAATTACACACGCTTAATAGCGACAACATCCTGGAGTAGCACATATAGCTACACAGAGAGAAATCCTTCTAAATAATATAGAATAAATTCTATACACCTTTTCTGTTGTGATAGAGATGAAATTTCCGATAATTTGAGCATAAGACACATTCAAACTAGTATGTGGAATCACTTTTTGTTTTCCTAAATAATTCTCTTGAGTTTTTCTCCCAGTGCACATATCTTTTAAGCTAAGAGCACCCATTCGCATGCTTACATAGGCGCATCAC

Sequences of four DNA constructs for creating new genetic variants, and three DNA fragments used for synthesizing probes for Southern blot are shown.

### Animal preparation

Experimental flies for both behavioral and imaging experiments were collected on the day of eclosion into vials containing standard cornmeal-based food and were kept as a group of up to 20 flies per vial at 25°C with 60% relative humidity and under a 9:00 AM to 9:00 PM light/dark cycle. Flies used in *shibere^ts^* experiments were kept at 18°C. Tester flies were transferred to an aluminum foil-covered vial with food containing 0.2 mm all-*trans* retinal (20 mm stock solution prepared in 95% ethanol; catalog #R2500, Millipore Sigma) 5–6 d before experimentation. Every 3 d, flies were transferred to vials containing fresh food. Tester flies were aged for 5–7 d if carrying *Otd-nls:FLPo*, and 14–16 d if carrying *fru^FLP^* to ensure consistent labeling of Tk-GAL4^FruM^ neurons (Asahina et al., 2014; Wohl et al., 2020). Rearing conditions of flies that carry *trans*-Tango elements are described below.

In behavioral experiments, a transgenic tester fly was paired with a target fly. Male target flies, wild-type Canton-S individuals (originally from the lab of Martin Heisenberg, University of Würzberg), were group reared with other males as virgins. To prepare mated female target flies, five Canton-S males were introduced into vials with 10 virgin 4-d-old females and were reared for 2 more days to let them mate. The males used for mating were discarded. At 3 d old, both male and mated female target flies were briefly anesthetized with CO_2_, and the tip of one of their wings was clipped with a razor blade to distinguish them from tester flies when tracking. This clipping treatment did not reduce the amount of lunging detected under our experimental settings (data not shown).

### Behavioral assays

Behavior assays were conducted in the evening (from 4:00 PM to 9:00 PM) at 22–25°C. For *shibere^ts^* experiments, flies were acclimated for 30 min at temperatures of 22 or 32°C before testing. These experiments were performed in a climate-controlled booth kept at 60% relative humidity.

Social behavior assays were performed in a 12-well acrylic chamber (Asahina et al., 2014) with food substrate (apple juice, Minute Maid) supplemented with 2.25% w/v agarose and 2.5% w/v sucrose (Hoyer et al., 2008) covering the entire arena floor. The wall was coated with Insect-a-Slip (catalog #2871C, BioQuip Products), and the ceiling was coated with SurfaSil Siliconizing Fluid (catalog #TS-42800, Thermo Fisher Scientific), to prevent flies from climbing as described previously (Hoyer et al., 2008; Asahina et al., 2014). Recording was done with USB3 digital cameras (Point Gray Flea3 USB 3.0, catalog #FL3-U3-13Y3M-C, Teledyne FLIR) controlled by BIAS acquisition software (IO Rodeo; https://github.com/iorodeo/bias). The camera was equipped with a machine vision lens (catalog #HF35HA1B, Fujinon) and an infrared long-pass filter (catalog #LP780-25.5, Midwest Optical Systems) to block light from the LED sources used for optogenetic neuronal activation (see below). Movies were taken at 60 frames per second in the AVI (Audio Movie 1 Interleave) format. Flies were discarded after each experiment. The food substrate was changed after five recordings.

Movie 1. Overexpression of tachykinin causes a male fly to attack a female target on optogenetic stimulation of Tk-GAL4^FruM^ neurons. 10.1523/JNEUROSCI.1734-22.2023.video.1

For optogenetic neuronal activation, a combined infrared (850 nm) and optogenetic (625 nm) LED backlight panel (described in https://www.janelia.org/open-science/combined-infrared-and-optogenetic-led-panel) was used as the light source. Briefly, the LED board was screwed to an aluminum heat sink (catalog #601403B06000, Aavid Thermalloy) with a nonconductive thermal pad wedged in between. Atop the board was a square wall of mirrors that faced inward with 114 mm sides × 25 mm height. This mirror box was designed to ensure that light collected toward the edges of the board were similar in power to that collected toward the center of the board where more LEDs were present. Two 13-mm-thick acrylic plates, separated by 6 mm, were placed above the backlight panel supported by 76 mm optical poles. The first of the two plates was translucent white, which evenly diffused the point source LEDs. An indicator infrared LED (850 nm) was placed above the first plate to report optogenetic LED stimulation, which was invisible in the recorded videos because of the long-pass filter installed in front of the camera. The second plate was clear; fly behavior chambers rested on it so that they were 25 mm above the LED board. To minimize red light exposure before experiments, overhead fluorescent lights were covered in blue cellophane (catalog #zprd_17968611a, JOANN Fabrics and Crafts). Additionally, a black box surrounded the arena and LED backlight panel to keep out light from surrounding experiments. An opening on top of the box allowed optical access by the camera as well as ambient light. It also had a small opening on one side to allow fly chambers to be moved in and out of the arena. The LED backlight panel was connected to a Teensy board, which interfaced with the flyBowl MATLAB custom code (provided by Yoshi Aso and Jinyang Liu, HHMI Janelia Research Campus) so that the LEDs used for optogenetics were synchronized with the BIAS encoding software.

### Quantification of social behavior data

Acquired movies were analyzed largely as described in (Ishii et al., 2020; Leng et al., 2020; Wohl et al., 2020). In brief, the movies were first processed by the FlyTracker program (Eyjolfsdottir et al., 2014; https://github.com/kristinbranson/FlyTracker). The number of lunges was quantified using behavioral classifiers developed in JAABA software (Kabra et al., 2013; https://sourceforge.net/projects/jaaba/files/), as described in Leng et al. (2020). The duration of time a tester fly orients toward a target fly (time orienting) was quantified as described previously (Ishii et al., 2020; Wohl et al., 2020). The distance traveled by a fly was calculated directly from the trx.mat file created by FlyTracker. The frame in which the infrared indicator LED turned on during the first LED stimulation period was used to align frames of movies.

### Immunohistochemistry

The following antibodies were used for immunohistochemistry with dilution ratios as indicated: rabbit anti-DsRed (1:1000; catalog #632496, Takara Bio; RRID:AB_10013483), mouse anti-BRP (1:100; catalog #nc82, concentrated, Developmental Studies Hybridoma Bank; RRID:AB_2314866), chicken anti-GFP (1:1000; catalog #ab13970, Abcam; RRID:AB_300798), rat anti-HA (1:1000; catalog #11867423001, Roche; RRID:AB_390918), goat anti-chicken Alexa 488 (1:100; catalog #A11039, Thermo Fisher Scientific; RRID:AB_2534096), goat anti-rat Alexa 488 (1:100, catalog #A11006, Thermo Fisher Scientific; RRID:AB_2534074), goat anti-rabbit Alexa 568 (1:100; catalog #A11036, Molecular Probes; RRID:AB_10563566), and goat anti-mouse Alexa 633 (1:100; catalog #A21052, Thermo Fisher Scientific; RRID:AB_2535719).

Immunohistochemistry of fly brains followed the protocol described in (Ishii et al., 2020; Wohl et al., 2020). Z-stack images were acquired by FV-1000 confocal microscopy (Olympus America) and were processed with Fiji software (Schindelin et al., 2012; RRID:SCR_002285; https://fiji.sc/). Minimum and maximum intensity thresholds were adjusted for enhanced clarity. Registration of brains to the JRC2018 INTERSEX template brain (Bogovic et al., 2020) was performed as described (Jefferis et al., 2007; Ishii et al., 2020; Wohl et al., 2020).

*Trans*-Tango flies used for immunohistochemistry were reared for 28–30 d at 21°C to allow sufficient expression of reporters in downstream areas with a maximal signal-to-noise ratio (Talay et al., 2017). To restrict expression of the human glucagon ligand, necessary for reporter translocation, to Tk-GAL4^FruM^ neurons, *Tk-GAL4^1^* expression was limited by a *tubulin-FRT-GAL80-FRT-stop* transgene and *fru^FLP^*.

### Image segmentation and quantification

To quantify the immunohistochemical fluorescence intensity of Syt:GFP and DenMark, Tk-GAL4^FruM^ neurons were first segmented into the superior medial protocerebrum (SMP) projection, ring-adjacent region, and axonal tract based on the confocal image of reporter proteins that visualize the neuroanatomy of Tk-GAL4^FruM^ neurons (myr:tdTomato for Syt:GFP samples, and cytosolic GFP for DenMark). The 3D-rendered images of Tk-GAL4^FruM^ neurons were manually segmented using the Paint Brush function of FluoRender software (Wan et al., 2009) as previously described in (Ishii et al., 2020; Wohl et al., 2020). Each segmented domain was converted back to an 8-bit stacked TIFF image, and a binary mask for the entire stack was created by adjusting the threshold value (20–40 depending on the image quality) in ImageJ software. The average signal intensity within the given domain was calculated as [sum of signal intensity in pixels within the mask]/[total number of pixels within the mask].

Signal intensity of GCaMP6f immunohistochemical fluorescence of *TkR86C^LexA^* and *TkR99D^LexA^* neurons in the vicinity of Tk-GAL4^FruM^ neurons was calculated in a similar manner as above. The SMP projection and ring-adjacent region were segmented based on the confocal image of CsChrimson:tdTomato expressed in Tk-GAL4^FruM^ neurons.

### Functional imaging

On the day of the experiment, flies were briefly anesthetized on ice and mounted on a custom chamber using ultraviolet curing adhesive (Norland Optical Adhesive 63) to secure the head and thorax to a tin foil base. The proboscis was also dabbed with glue to prevent its extension from altering the position of the brain. The head cuticle was removed with sharp forceps in *Drosophila* adult hemolymph-like saline (Wang et al., 2003) at room temperature. After cuticle removal, the saline was exchanged with a fresh volume.

Optogenetic stimulation was applied with an external fiber-coupled LED of 625 nm (catalog #M625F2, Thorlabs) controlled by a programmable LED driver (catalog #DC2200, Thorlabs). The end of the LED fiber (catalog #M28L01, Thorlabs) was placed 5 mm from the brain. The LED produced 10 ms pulses 10 s at 0.5, 1, or 5 Hz. The energy from the LED that the neurons received was estimated from the measurement of the LED power as 0.2 mA using a photodiode power sensor (catalog #S130C, Thorlabs) coupled to a digital optical power/energy meter (catalog #PM100D, Thorlabs) 5 mm away from the end of the LED fiber.

The multiphoton laser scanning microscope (FV-MPE-RS, Olympus), equipped with 25× water immersion objective (catalog #XLPLN25XWMP2, Olympus), was used for monitoring the fluorescence of GCaMP6f. The recordings began 5–10 s before a 10 s stimulation and continued for 10–20 s after stimulation for a total of 25–40 s. GCaMP6f fluorescence was visualized with a tunable laser set at 920 nm output (Spectra-Physics InSight DL Dual-OL, Newport, and CsChrimson:tdTomato was visualized with an auxiliary laser with a fixed output of 1040 nm. Images were taken at 5–7 Hz, depending on the size of scanning area, with a 256 × 256 pixel resolution.

Acquired images (OIR format) were converted and analyzed in Fiji with the Olympus ImageJ plug-in (http://imagej.net/OlympusImageJPlugin). Imaging windows were chosen that maximally captured the Tk-GAL4^FruM^ neuronal projections in the SMP or in the ring-adjacent region using the fluorescence of CsChrimson:tdTomato. Polygonal regions of interest (ROIs) were drawn using the tdTomato fluorescence, and ΔF/F of GCaMP6f was calculated using a custom-written MATLAB code. First, the baseline fluorescence value (*F*_base_) was calculated by averaging the fluorescence for 5 s preceding the stimulation. ΔF/F for each frame ((ΔF/F)_frame=N_) was calculated as follows:

$${\left(\Delta \text{F}/\text{F}\right)}_{\text{frame}=\text{N}}=({\text{F}}_{\text{frame}=\text{N}}–{\text{F}}_{\text{base}})/{\text{F}}_{\text{base}}.$$

Then, the ΔF/F_frame=N_ for frames taken during the 10 s LED stimulation were averaged to calculate the ΔF/F of a given trial. Frames that contained LED light for optogenetic stimulation were excluded from the analysis. Values from one to five trials were averaged for each condition. Trials with excessive movement were discarded.

Our preliminary study indicated that baseline fluorescence of the *QUAS-GCaMP3* transgene (stock #52231, BDSC) driven by *trans*-Tango was not sufficient to be visualized under two-photon microscopy. Thus, we constructed *15XQUAS-IVS-Syn21-GCaMP6f-p10* (see above for details) and used two copies of the insertions for *trans*-Tango imaging experiments. These flies were transferred to 0.2 mm all-*trans*-retinal food 6 d before experimentation. Because of the higher level of expression of our GCaMP6f constructs, we needed to age flies only for 16–20 d.

### Pharmacology

A 2.5 mm master solution of mecamylamine was made by dissolving mecamylamine hydrochloride (catalog #M9020, Millipore Sigma) in *Drosophia* adult hemolymph saline (Wang et al., 2003). Pretreatment trials were recorded first. Then mecamylamine saline was added to the imaging saline reservoir for a final concentration of 25 μm via pipetting. The drug-infused saline was then gently mixed. For vehicle experiments, the same amount of saline was added but without mecamylamine. Imaging resumed 15 min after adding the solution. When treatment trials were complete, washout of drug was performed in the following steps. First, the saline, with or without mecamylamine, was replaced with drug-free saline six times. Fifteen minutes later, the saline was again replaced twice. Calcium imaging for the washout condition resumed 15 min after the second wash cycle so that it began a total of ∼30 min after the first washing.

### Experimental design and statistical analysis

Male flies were used in all experiments. The sample number for each experiment is shown either in a figure or in the figure legend. Unless otherwise noted (see Figs. 2*D*,*E*,*G*,*H*,*O*,*Q*, 4*L*, 9*E*,*F*), one data point was measured from an independent animal. Experiments were not blinded to animal genotypes, optogenetic stimulation conditions, temperature, or pharmacological conditions, but measurements of behavior and drawing of ROIs (for quantifying fluorescence) used a computational process that was blind to the sample identity (see above, “Quantification of social behavior data” and “Functional imaging”). No statistical method was used to predetermine sample size before the study.

Statistical analyses were conducted using MATLAB, with two exceptions. First, 95% confidence intervals were calculated using Microsoft Excel CONFIDENCE.T function. Second, repeated-measures ANOVA was performed using Prism 9.4.1 (GraphPad).

**Figure 1.**
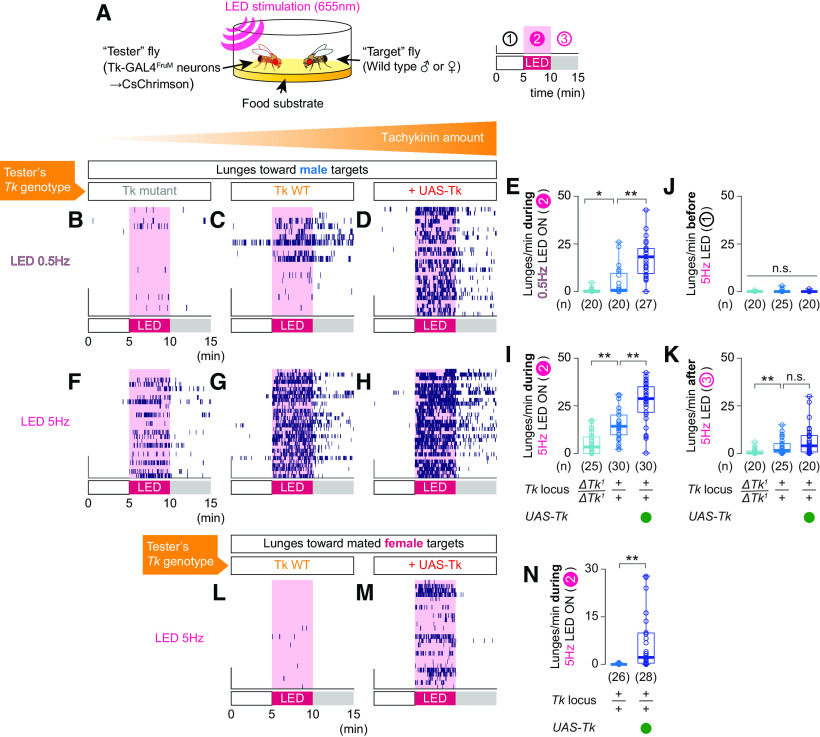
Tachykinin amount controls the intensity of aggression induced by optogenetic activation of Tk-GAL4^FruM^ neurons. ***A***, Design of the optogenetic behavioral assay. ***B–M***, Raster plots of lunges toward a male (***B–D***, ***F–H***) or a mated female (***L***, ***M***) target fly induced by optogenetic activation of Tk-GAL4^FruM^ neurons, either at 0.5 Hz (***B–D***) or at 5 Hz (***F–H***, ***L***, ***M***). Box plots of lunges targeted toward male (***E***, ***I–K***) and female (***H***), before (***J***), during (***E***, ***I***, ***N***), or after (***K***) optogenetic stimulation of Tk- GAL4^FruM^ neurons. In ***E***, ***I***, ***K***, **p < 0.01, n.s. *p* > 0.05 by Kruskal–Wallis one-way ANOVA and *post hoc* Mann–Whitney *U* test. In ***J***, n.s. *p* > 0.05 by Kruskal–Wallis one-way ANOVA. In ***N***, **p < 0.01 by Mann–Whitney *U* test. For ***E*** and ***H***, see Extended Data 1 for the complete data and statistical results. ***B*** and ***F*** are data from *Tk* null mutants; ***C***, ***G***, and ***L*** are data from *Tk* wild type; and ***D***, ***H***, and ***M*** are data from animals with a *UAS-Tk* transgene. In ***E***, ***I–K***, and ***N***, the *Tk* genotypes are indicated at the bottom of ***I*** and ***K***. For ***E***, ***I–K***, ***N***, Table 9 and Extended Data 1 contain the complete data and statistical results.

The complete experimental design and statistical results are described in Table 9. All source data are presented in Extended Data 1. Nonparametric analyses were used for behavioral data (Fig. 1*E*,*I–K*,*N*; see Figs. 6*C–E*,*G*, 8*J*,*K*) except where Fisher's exact test was used (see Fig. 11*D*). After behaviors within each time window were calculated, the Kruskal–Wallis one-way ANOVA (kruskalwallis) was used to evaluate whether a given behavior was significantly different among >2 different genotypes. When the *p* value was below 0.05, the *post hoc* Mann–Whitney *U* test (ranksum) with Bonferroni correction for multiple comparison was used to detect significant differences between the tester and control genotypes. When the uncorrected *p* value was <0.05 but did not pass the critical (α) value, the uncorrected value is shown in figures in parentheses. ANOVA was omitted when the comparison was between two different genotypes (Fig. 1*N*; see Fig. 8*K*). Except where the percentage of lunging tester flies are shown (see Fig. 11*D*), all behavioral data are presented in box plots with individual data points.

**Table 9.** Summary statistics table

Figure	Test type	Degrees of freedom	Name of test statistic	Statistic value	*p* Value, uncorrected	α Value, correction with Bonferroni if multiple comparison was applied	Program and function used
Figure 1							
1 *E*							
Across genotypes	Kruskal–Wallis one-way ANOVA	3	χ^2^	42.3376468	**3.40E-09** *^b^*	0.05	MATLAB, kruskalwallis
*Post hoc*							
*Tk* mutants vs wild type	Mann–Whitney *U* test	NA	*z*	2.428124112	***0.015177149*** *^a^*	0.016666667	MATLAB, ranksum
*Tk* heterozygotes vs wild type	Mann–Whitney *U* test	NA	*z*	1.337605367	0.181025114*^b^*	0.016666667	MATLAB, ranksum
*Tk* wild type vs +UAS-Tk	Mann–Whitney *U* test	NA	*z*	−3.703391686	**2.13E-04**	0.016666667	MATLAB, ranksum
1 *J*							
Across genotypes	Kruskal–Wallis one-way ANOVA	2	χ^2^	1.486111196	0.475658272	0.05	MATLAB, kruskalwallis
1 *I*							
Across genotypes	Kruskal–Wallis one-way ANOVA	2	χ^2^	44.44413204	**2.23E-10** *^b^*	0.05	MATLAB, kruskalwallis
*Post hoc*							
*Tk* heterozygotes vs wild type	Mann–Whitney *U* test	NA	*z*	−4.5459	**5.47E-06** *^b^*	0.025	MATLAB, ranksum
*Tk wild type* vs+*UAS-Tk*	Mann–Whitney *U* test	NA	*z*	4.0292	**5.60E-05** *^b^*	0.025	MATLAB, ranksum
1 *K*							
Across genotypes	Kruskal–Wallis one-way ANOVA	2	χ^2^	18.63718634	**8.97E-05** *^b^*	0.05	MATLAB, kruskalwallis
1 *N*							
*Tk wild type* vs +*UAS-Tk*	Mann–Whitney *U* test	NA	*z*	5.141555531	**2.72E-07** *^b^*	0.05	MATLAB, ranksum
Figure 2							
2 *D*							
Across brain regions	Repeated-measures ANOVA	DFn = 1.041, DFd = 7.285	*f*	81.68	**4.15E-05** *^b^*	0.05	Prism, sphericity not assumed
*Post hoc*							
Commisural tract–SMP	Paired *t* test	7	*t*	1.73771339	1.26E-01*^b^*	0.016666667	MATLAB, *t* test
Commisural tract–ring adjacent	Paired *t* test	7	*t*	9.543679954	**2.91E-05** *^b^*	0.016666667	MATLAB, *t* test
SMP–ring adjacent	Paired *t* test	7	*t*	8.695687463	**5.33E-05**	0.016666667	MATLAB, *t* test
2 *E*							
SMP–ring adjacent	Paired *t* test	7	*t*	19.48695662	**2.34E-07** *^b^*	0.05	MATLAB, *t* test
2 *G*							
Across brain regions	Repeated Measures ANOVA	DFn = 1.363, DFd = 14.99	*f*	27.06	**1.34E-04** *^b^*	0.05	Prism, sphericity not assumed
*Post hoc*							
Commisural tract–SMP	Paired *t* test	11	*t*	5.165816824	**3.11E-04** *^b^*	0.016666667	MATLAB, *t* test
Commisural tract–ring adjacent	Paired *t* test	11	*t*	0.903584977	3.86E-01*^b^*	0.016666667	MATLAB, *t* test
SMP–ring adjacent	Paired *t* test	11	*t*	−6.850926008	**2.76E-05** *^b^*	0.016666667	MATLAB, *t* test
2 *H*							
SMP–ring adjacent	Paired *t* test	11	*t*	−5.730345391	**1.32E-04**	0.05	MATLAB, *t* test
2 *Q*							
SMP–ring adjacent	Paired *t* test	5	*t*	−4.475072015	**6.55E-03** *^b^*	0.05	MATLAB, *t* test
Figure 5							
5 *E_2_*							
Across treatment	Repeated-measures ANOVA	DFn = 1.45, DFd = 14.5	*f*	32.91	**5.14E-05** *^b^*	0.05	Prism, sphericity not assumed
*Post hoc*							
Pre vs during	Paired *t* test	10	*t*	−6.846388397	**4.48E-05** *^b^*	0.025	MATLAB, *t* test
During vs post	Paired *t* test	10	*t*	−5.024161811	**5.19E-04** *^b^*	0.025	MATLAB, *t* test
5 *F_2_*							
Across treatment	Repeated-measures ANOVA	DFn = 1.902, DFd = 15.21	*f*	0.6431	0.5318	0.05	Prism, sphericity not assumed
Figure 6							
6 *C*							
Across genotypes	Kruskal–Wallis one-way ANOVA	4	χ^2^	62.64932452	**8.04E-13** *^b^*	0.05	MATLAB, kruskalwallis
*Post hoc*							
Genotype [1] vs genotype [2]	Mann–Whitney *U* test	NA	*z*	4.149405565	**3.33E-05** *^b^*	0.0125	MATLAB, ranksu
Genotype [1] vs genotype [3]	Mann–Whitney *U* test	NA	*z*	3.613396039	**3.02E-04** *^b^*	0.0125	MATLAB, ranksum
Genotype [1] vs genotype [4]	Mann–Whitney *U* test	NA	*z*	−2.555593377	**1.06E-02** *^a^*	0.0125	MATLAB, ranksum
Genotype [1] vs genotype [5]	Mann–Whitney *U* test	NA	*z*	−2.029280015	4.24E-02	0.0125	MATLAB, ranksum
6 *D*							
Across genotypes	Kruskal–Wallis one-way ANOVA	4	χ^2^	9.646611365	**4.68E-02** *^a^*	0.05	MATLAB, kruskalwallis
*Post hoc*							
Genotype [1] vs genotype [2]	Mann–Whitney *U* test	NA	*z*	−0.269876839	0.787255006	0.0125	MATLAB, ranksum
Genotype [1] vs genotype [3]	Mann–Whitney *U* test	NA	*z*	0.68781414	0.4916	0.0125	MATLAB, ranksum
Genotype [1] vs genotype [4]	Mann–Whitney *U* test	NA	*z*	−1.769890059	0.076745457	0.0125	MATLAB, ranksum
Genotype [1] vs genotype [5]	Mann–Whitney *U* test	NA	*z*	−1.835685229	0.066404224	0.0125	MATLAB, ranksum
6 *E*							
Across genotypes	Kruskal–Wallis one-way ANOVA	4	χ^2^	79.40956975	**2.32E-16** *^b^*	0.05	MATLAB, kruskalwallis
*Post hoc*							
Genotype [1] vs genotype [2]	Mann–Whitney *U* test	NA	*z*	4.035600179	**5.45E-05** *^b^*	0.0125	MATLAB, 'ranksum'
Genotype [1] vs genotype [3]	Mann–Whitney *U* test	NA	*z*	3.897613459	**9.71E-05** *^b^*	0.0125	MATLAB, 'anksum
Genotype [1] vs genotype [4]	Mann–Whitney *U* test	NA	*z*	5.020171433	**5.16E-07** *^b^*	0.0125	MATLAB, ranksum
Genotype [1] vs genotype [5]	Mann–Whitney *U* test	NA	*z*	−4.217470365	**2.47E-05** *^b^*	0.0125	MATLAB, ranksum
6 *G*							
Across genotypes	Kruskal–Wallis one-way ANOVA	4	χ^2^	87.54663635	**4.37E-18** *^b^*	0.05	MATLAB, kruskalwallis
*Post hoc*							
Genotype [1] vs genotype [2]	Mann–Whitney *U* test	NA	*z*	2.113774547	**3.45E-02**	0.0125	MATLAB, ranksum
Genotype [1] vs genotype [3]	Mann–Whitney *U* test	NA	*z*	1.5291119	0.126236706	0.0125	MATLAB, ranksum
Genotype [1] vs genotype [4]	Mann–Whitney *U* test	NA	*z*	−4.898261068	**9.67E-07** *^b^*	0.0125	MATLAB, ranksum
Genotype [1] vs genotype [5]	Mann–Whitney *U* test	NA	*z*	−4.90912553	**9.15E-07** *^b^*	0.0125	MATLAB, ranksum
Figure 8							
8 *D_1_*							
Across genotypes	One-way ANOVA	2	*f*	7.103958365	**2.64E-03** *^a^*	0.05	MATLAB, anova1
*Post hoc*							
*Tk* mutants vs wild type	Upaired *t* test	20	*t*	2.75418248	**1.22E-02** *^a^*	0.025	MATLAB, ttest2
*Tk wild type vs.* +*UAS-Tk*	Upaired *t* test	26	*t*	−1.586354175	1.25E-01	0.025	MATLAB, ttest2
8 *D_2_*							
Across genotypes	One-way ANOVA	2	*f*	7.79642538	9.77E-04*^b^*	0.05	MATLAB, anova1
*Post hoc*							
*Tk* mutants vs wild type	Upaired *t* test	38	*t*	3.442073309	**1.42E-03** *^b^*	0.025	MATLAB, ttest2
*Tk wild type* vs +*UAS-Tk*	Upaired *t* test	43	*t*	0.053124517	0.957878742	0.025	MATLAB, ttest2
8 *D_3_*							
Across genotypes	One-way ANOVA	2	*f*	4.359677873	1.71E-02*^a^*	0.05	MATLAB, anova1
*Post hoc*							
*Tk* mutants vs wild type	Upaired *t* test	38	*t*	2.857619465	**6.89E-02** *^b^*	0.025	MATLAB, ttest2
*Tk wild type* vs +*UAS-Tk*	Upaired *t* test	43	*t*	1.019738906	0.313555579	0.025	MATLAB, ttest2
8 *H*							
Across genotypes	One-way ANOVA	2	*f*	2.02E-01	0.818157731	0.05	MATLAB, anova1
8 *J*							
Across genotypes	Kruskal–Wallis one-way ANOVA	5	χ^2^	1.15E + 02	**4.53E-23** *^b^*	0.05	MATLAB, kruskalwallis
*Post hoc*							
Genotype [6] vs genotype [1]	Mann–Whitney *U* test	NA	*z*	6.31E + 00	**2.74E-10** *^b^*	0.01	MATLAB, ranksum
Genotype [6] vs genotype [2]	Mann–Whitney *U* test	NA	*z*	6.40E + 00	**1.51E-10** *^b^*	0.01	MATLAB, ranksum
Genotype [6] vs genotype [3]	Mann–Whitney *U* test	NA	*z*	6.24E + 00	**4.29E-10** *^b^*	0.01	MATLAB, ranksum
Genotype [6] vs genotype [4]	Mann–Whitney *U* test	NA	*z*	−1.291834131	0.196414593	0.01	MATLAB, ranksum
Genotype [6] vs genotype [5]	Mann–Whitney *U* test	NA	*z*	−1.18148639	0.237409557	0.01	MATLAB, ranksum
8 *K*							
Imaging genotype, *Tk* wild type vs +UAS-Tk	Mann–Whitney *U* test	NA	*z*	5.029640711	**4.91E-07** *^b^*	0.05	MATLAB, ranksum
Figure 9 *F*							
SMP–ring adjacent	Paired *t* test	7	*t*	−7.57E-01	0.473676454	0.05	MATLAB, *t* test
Figure 10							
10 *I*							
*Tk wild type* vs +*UAS-Tk*	Upaired *t* test	17	*t*	1.253061473	2.27E-01	0.05	MATLAB, ttest2
10 *J*							
*Tk wild type* vs +*UAS-Tk*	Upaired *t* test	17	*t*	3.32E + 00	**4.07E-03** *^b^*	0.05	MATLAB, ttest2
Figure 11 *D*							
Genotype [1] vs genotype [2]	Fisher's exact test	NA	NA	NA	**4.97E-04** *^b^*	0.016666667	MATLAB, fishertest
Genotype [1] vs genotype [3]	Fisher's exact test	NA	NA	NA	**3.77E-04** *^b^*	0.016666667	MATLAB, fishertest
Genotype [1] vs genotype [4]	Fisher's exact test	NA	NA	NA	**3.79E-05** *^b^*	0.016666667	MATLAB, fishertest

This table contains type of statistical test, degree of freedom, and other statistics-specific values used in each figure.

*^a^*: *p* < 0.05,

*^b^*: *p* < 0.01 or equivalent after multiple comparison where applicable.

**Figure 2.**
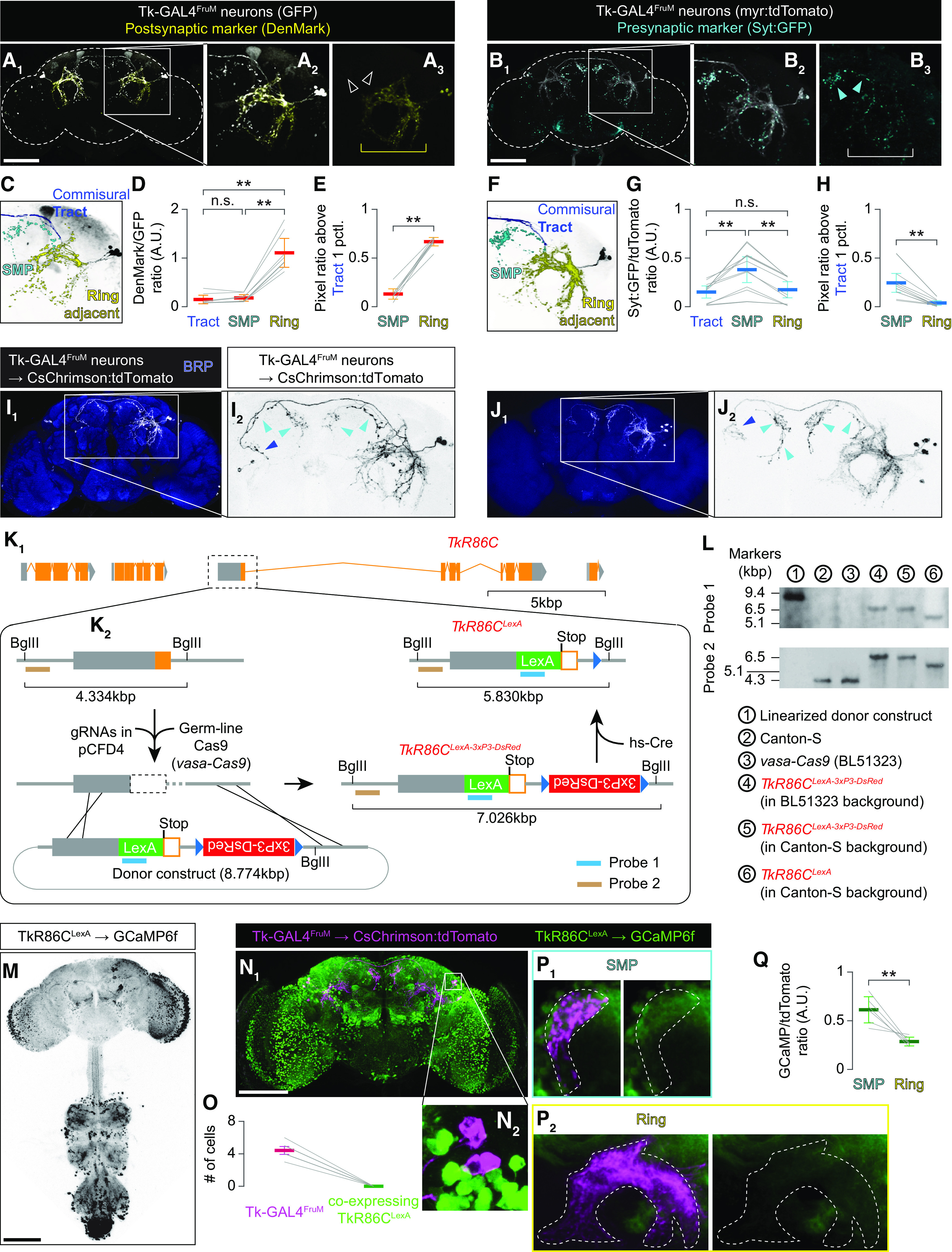
Tk-GAL4^FruM^ neurons are positioned to make synaptic contact with *TkR86C^LexA^* neurons. ***A_1–3_***, A representative image of a male brain expressing GFP (white) and the postsynaptic marker DenMark (yellow), which is present in the ring-adjacent region (***A_3_***, yellow bracket) but not in the projection to the SMP (empty white arrowheads). Scale bar, 100 µm. ***B_1–3_***, Representative image of a male brain expressing myristoylated tdTomato (myr:tdTomato, white) and the presynaptic marker synaptotagmin:GFP (Syt:GFP, cyan), which is present in the projection to the SMP (***B_3_***, cyan arrowheads) but only sparsely observed in the ring-adjacent region (white bracket). Scale bar, 100 µm. ***C***, ***F***, Segmentation of the region shown in ***A_2_*** (***C***) and ***B_2_*** (***F***). ***D***, ***G***, DenMark (***D***, *n* = 8 hemibrains from 4 brains) and Syt:GFP (***G***, *n* = 12 hemibrains from 6 brains) immunohistochemical signals relative to GFP (***D***) and myr:tdTomato (***G***) signals in the commissural tract (Tract), SMP, and ring-adjacent region (Ring); **p < 0.01, n.s. *p* > 0.05 by repeated-measures ANOVA and *post hoc* paired *t* test. ***E***, ***H***, DenMark (***E***, *n* = 8) and Syt:GFP (***H***, *n* = 12) immunohistochemical signals in SMP and ring areas relative to the signals in the tract; ** *p* < 0.01 by paired *t* test. ***I_1_***–***J_2_***, Two independent samples of unilaterally labeled Tk-GAL4^FruM^ neurons are shown. These were generated by stochastic inactivity of fru^FLP^ on one side of the brain in animals that also carried *Tk-GAL4^1^* and *20XUAS*>*myr:TopHAT2*>*CsChrimson:tdTomato* (in attP2) transgenes. Magnified images of Tk-GAL4^FruM^ neurons in ***I_1_*** and ***J_1_*** are shown in ***I_2_*** and ***J_2_***, respectively. Cyan arrowheads indicate bouton-like varicosities in the SMP that emanate from the putative axon tract, which crosses the midline and reaches the area where the Tk-GAL4^FruM^ neurons in the contralateral side extend their dendritic arbors (blue arrowheads). ***K_1_*_,_*_2_***, A schematic of the steps taken to create the *TkR86C^LexA^* allele. The first exon of *TkR86C* was modified (***K_1_***) with CRISPR/Cas9-mediated homologous recombination as described in ***K_2_***. ***L***, Southern blotting analysis of *TkR86C^LexA^* alleles. Probe 1 targets upstream of the *TkR86C* locus, whereas probe 2 targets the coding region of LexA (***K_2_***). Note that flies 4–6 were homozygous for the *TkR86C* alleles. ***M***, Representative expression pattern of GCaMP6f driven by *TkR86C^LexA^* in the nervous system, visualized by immunohistochemistry. Scale bar, 100 µm. ***N***, A representative image of a male brain expressing GCaMP6f driven by *TkR86C^LexA^* (green) and CsChrimson:tdTomato under intersectional control of *Tk-GAL4^1^* and *fru^FLP^* (magenta). Scale bar, 100 µm. ***O***, *TkR86C^LexA^* does not label Tk-GAL4^FruM^ neurons (*n* = 12 hemibrains from 6 brains). ***P_1_***,***_2_***, Distribution of immunohistochemical signals of GCaMP6f driven by *TkR86C^LexA^* (green), and CsChrimson:tdTomato under intersectional control of *Tk-GAL4^1^* and *fru^FLP^* (magenta). The magnified images near the Tk-GAL4^FruM^ neuronal projections (dashed white lines) in the SMP (***P_1_***) and the ring region (***P_2_***) from an averaged image stack of eight standardized hemibrains (see above, Materials and Methods, Image segmentation and quantification) are shown. ***Q***, Average GCaMP6f immunohistochemical fluorescence in the SMP and ring-adjacent region as defined by CsChrimson:tdTomato immunohistochemical signals in Tk-GAL4^FruM^ neurons (*n* = 6 hemibrains from 3 brains); ** *p* < 0.01 by paired *t* test. The thick line and error bars in ***D***, ***E***, ***G***, ***K*, *H***, and **Q** represent the average and 95% confidence intervals. Table 9 and Extended Data 1 contain the complete data and statistical results.

Fluorescence data from immunohistochemical (Fig. 2*D*,*E*,*G*,*H*,*O*,*Q*; see Figs. 5*A3–C3*, 9*E*,*F*) and functional imaging data (see Figs. 5*E2*,*F2*, 8*D*,*H*, 10*I*,*J*) were analyzed using parametric tests. Datasets from more than two independent sources (e.g., different genotypes) were first analyzed with one-way ANOVA (anova1). When the *p* value was below 0.05, the *post hoc* Welch's *t* test (ttest2) with Bonferroni correction was used to detect significant differences between genotypes or conditions. Datasets from more than two balanced sources (Fig. 2*D*,*G*; see Fig. 5*E2*,*F2*) were first analyzed with GraphPad Prism repeated-measures ANOVA. When the *p* value was below 0.05, the *post hoc* paired *t* test (*t* test) with Bonferroni correction was used to detect significant differences between measurements. ANOVA was omitted when comparing two datasets (Fig. 2*E*,*H*,*Q*; see Figs. 9*F*, 10*I*,*J*). All fluorescence data were presented as mean ± 95% confidence intervals with individual data points.

All data points, statistical results, and (for parametric tests) 95% confidence intervals are presented in the Extended Data 1.

Further information and requests for resources and reagents should be directed to and will be fulfilled by lead author Kenta Asahina at kasahina@salk.edu.

## Results

### Tachykinins in Tk-GAL4^FruM^ neurons quantitatively and qualitatively enhance aggression

Tk-GAL4^FruM^ neurons promote aggression toward other males but not toward females, likely because of a *doublesex* (*dsx*)-dependent mechanism that enforces target specificity of male aggression (Wohl et al., 2020). Previous work that used a thermogenetic approach did not address whether tachykinin released from Tk-GAL4^FruM^ neurons can alter the target sex specificity of male aggression (Asahina et al., 2014). Here, we quantified male- and female-directed aggressive behavior induced by optogenetic activation of Tk-GAL4^FruM^ neurons by the red-shifted channelrhodopsin CsChrimson (Klapoetke et al., 2014) while varying the level of tachykinin expression in these neurons (Fig. 1*A*).

10.1523/JNEUROSCI.1734-22.2023.ed1 Extended Data 1 This figure contains raw data that were used to create all figures. Details of statistical results are also included. Download Extended Data 1, XLSX file.

Consistent with the results from thermogenetic manipulation, the tachykinin null mutation attenuated male-directed aggression induced by optogenetic activation of Tk-GAL4^FruM^ neurons, whereas overexpression of tachykinin in Tk-GAL4^FruM^neurons enhanced male-directed aggression at two different stimulation frequencies (Fig. 1*B–I*). Aggression levels were comparably low among genotypes during the prestimulation time windows (Fig. 1*F–H*,*J*), suggesting that tachykinin needs to be released in an activity-dependent manner to promote aggression. Also, overexpression of tachykinin did not increase persistent aggression in the poststimulus time window (Fig. 1*H*,*K*), further arguing that tachykinin promotes aggression by enhancing the immediate physiological impact of Tk-GAL4^FruM^ neuronal activity on the circuit.

Intriguingly, overexpression of tachykinin caused male tester flies to attack female targets during optogenetic stimulation, which was rare in wild-type flies (Fig. 1*L–N*; Fernández et al., 2010; Monyak et al., 2021). Such qualitative enhancement of aggression may be mediated by recruitment of a new circuit component. These results suggest that tachykinin from Tk-GAL4^FruM^ neurons is involved in both quantitative (toward males) and qualitative (toward females) enhancement of male aggressive behavior.

### Anatomical relationship between *TkR86C*-expressing neurons and Tk-GAL4^FruM^ neurons

To begin elucidating the downstream targets of Tk-GAL4^FruM^ neurons, we first needed to identify which arborizations were dendritic and which were axonal. We used the genetically encoded postsynaptic marker DenMark (Nicolaï et al., 2010) to identify dendrites and the presynaptic marker synaptotagmin:GFP (Syt:GFP; Zhang et al., 2002) to identify axon terminals of Tk-GAL4^FruM^ neurons. Postsynaptic (dendritic) markers were primarily detected in arborizations in the lateral crescent, ring, and lateral junction structures (Cachero et al., 2010; Yu et al., 2010; Fig. 2*A1*), which are proposed to integrate olfactory and gustatory information (Yu et al., 2010; Clowney et al., 2015; Auer and Benton, 2016). On the other hand, presynaptic markers were primarily detected in the branches projecting to the SMP and in the bilateral arch (Fig. 2*B1*) (Yu et al., 2010; Ito et al., 2014; Fig. 2*B*), which were largely devoid of DenMark signal (Fig. 2*A2*,*3*). Both presynaptic and postsynaptic markers were mostly undetectable in the commissural tract that extends from the dorsal side of the lateral junction (Fig. 2*A2*,*3*,*B2*,*3*). The Syt:GFP-enriched branches to the SMP emanate from this tract, suggesting that it is the axonal tract of Tk-GAL4^FruM^ neurons.

To quantify these observations, we segmented Tk-GAL4^FruM^ neurons into three domains: arborizations in the SMP, arborizations in lateral regions (hereafter called “ring-adjacent” regions), and the commissural tracts (Fig. 2*C*,*F*). We then measured the average signal intensity of both Syt:GFP and DenMark within each domain. As expected, DenMark signals were enriched in the ring-adjacent region (Fig. 2*D*,*E*), whereas Syt:GFP signals were enriched in the SMP projection (Fig. 2*G*,*H*). Punctated Syt:GFP signals were also sparsely detected in regions of the Tk-GAL4^FruM^ neurons enriched with DenMark signals (Fig. 2*B2*,*3*). At least some of this Syt:GFP signal likely belongs to presynaptic termini from the contralateral projection. Samples from brains with Tk-GAL4^FruM^ neurons labeled unilaterally show that the axonal commissural tract crosses the midline and projects to a medial part of the ring on the contralateral side (Fig. 2*I*,*J*). It is also possible that the ring-adjacent region contains presynaptic sites that mediate retrograde or dendrodendritic communications. Overall, these largely segregated distributions of presynaptic and postsynaptic markers suggest that neurotransmitters from Tk-GAL4^FruM^ neurons are mainly released in the SMP.

Previous work showed that mutation of the tachykinin receptor gene *TkR86C* attenuates aggression triggered by thermogenetic excitation of Tk-GAL4^FruM^ neurons (Asahina et al., 2014). This suggests that at least a subset of the circuit downstream of Tk-GAL4^FruM^ neurons expresses *TkR86C*. To visualize these putative downstream neurons, we created a novel knock-in allele of *TkR86C*, named *TkR86C^LexA^*, using CRISPR/Cas9-mediated gene editing (Gratz et al., 2014; Fig. 2*K*,*L*). *TkR86C^LexA^*-expressing neurons were numerous and widespread (visualized with immunohistochemistry against LexA-driven GCaMP6f; Chen et al., 2013), both in the central brain and in the ventral nerve cord (Fig. 2*M*). This expression pattern is similar to that of a previously reported *TkR86C* knock-in allele (Kondo et al., 2020). The *TkR86C^LexA^* expression pattern is also consistent with the broad expression of tachykinin peptides (Winther et al., 2003). Importantly, Tk-GAL4^FruM^ neurons do not express *TkR86C^LexA^* (Fig. 2*N*,*O*), suggesting that tachykininergic modulation by Tk-GAL4^FruM^ neurons through *TkR86C* does not employ an autocrine mechanism (Choi et al., 2012).

We next asked whether *TkR86C*-expressing neurons and Tk-GAL4^FruM^ neurons are directly connected by examining the anatomic relationship between these two neuronal populations. Immunohistochemistry revealed that the presynaptic regions of Tk-GAL4^FruM^ neurons in the SMP are in close proximity to the neuronal processes of *TkR86C^LexA^* neurons (Fig. 2*P1*). In contrast, *TkR86C^LexA^* neurons showed less overlap with the postsynaptic ring-adjacent regions of Tk-GAL4^FruM^ neurons (Fig. 2*P2*,*Q*). This suggests that some *TkR86C^LexA^* neurons are positioned to receive synaptic inputs in the SMP from Tk-GAL4^FruM^ neurons.

### *TkR86C*-expressing neurons are functionally downstream of Tk-GAL4^FruM^ neurons

We next sought to obtain physiological evidence that *TkR86C^LexA^* neurons receive neural input from Tk-GAL4^FruM^ neurons. The anatomic results thus far are consistent with the idea that a subset of *TkR86C^LexA^* neurons is synaptically downstream of Tk-GAL4^FruM^ neurons. However, the mere proximity of neurites does not guarantee the presence of synapses. Moreover, although some studies have observed peptide-containing dense core vesicles primarily near presynaptic sites (Jan et al., 1980; Salio et al., 2006; Schlegel et al., 2016; Tao et al., 2018), neuropeptides are also released extrasynaptically (Jan and Jan, 1982; Karhunen et al., 2001) and affect the physiology of target neurons that are not synaptically connected (Jan et al., 1980; Jan and Jan, 1982; Nässel, 2009; van den Pol, 2012). To determine whether *TkR86C^LexA^* neurons receive neural input from Tk-GAL4^FruM^ neurons near their synaptic termini or in extrasynaptic locations, we visualized *TkR86C^LexA^* neuronal activity patterns across a large portion of the brain in response to optogenetic excitation of Tk-GAL4^FruM^ neurons.

**Figure 3.**
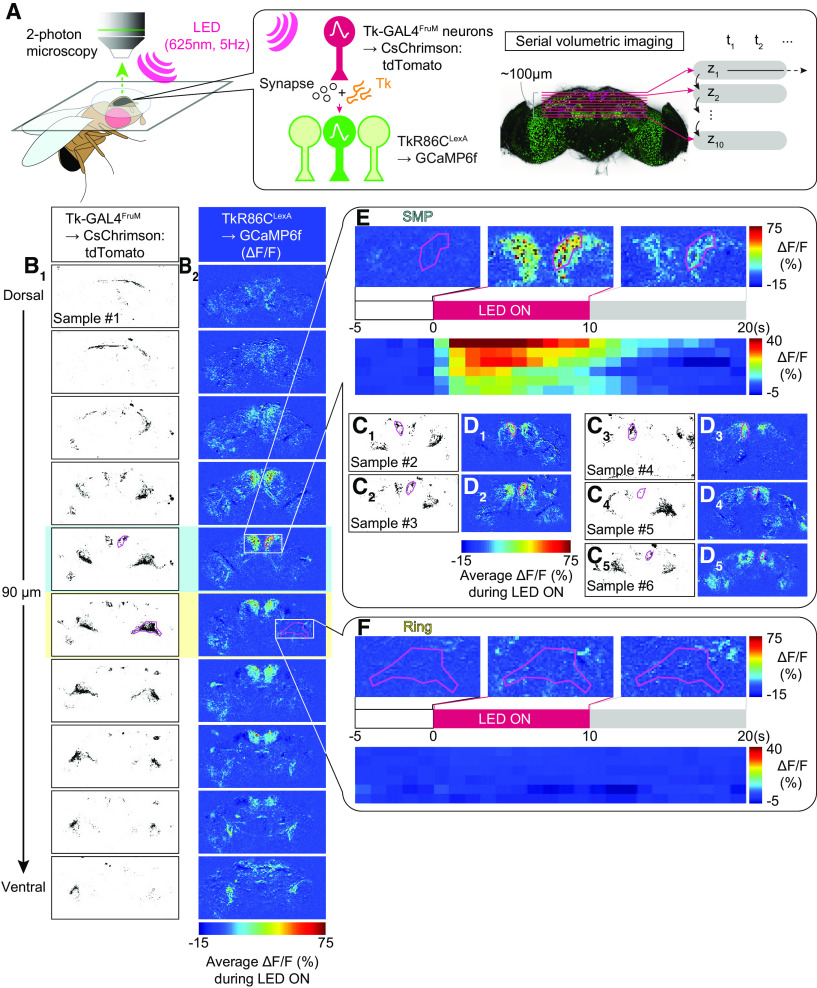
*TkR86C^LexA^* neurons are consistently activated by Tk-GAL4^FruM^ neurons. ***A***, A schematic representation of the functional serial volume imaging. ***B_1_–F***, Images of CsChrimson:tdTomato fluorescence in Tk-GAL4^FruM^ neurons (***B_1_***, ***C^1-5^***) and the average fluorescence of GCaMP6f in *TkR86C^LexA^* neurons (***B_2_***, ***D^1-5^***). FThe GCaMP6f signals are shown in pseudocolor as the relative increase in fluorescence (ΔF/F) during optogenetic activation of Tk-GAL4^FruM^ neurons. *Z*-series of images are shown for a representative sample (***B^1-5^***), whereas images that contain the projections of Tk-GAL4^FruM^ neurons to the SMP are shown for the other five samples (***C^1-5^*, *D^1-5^***). ***E***, ***F***, Pseudocolored GCaMP6f fluorescence changes (ΔF/F) in the vicinity of the projections of Tk-GAL4^FruM^ neurons to the SMP (***E***, highlighted with cyan in ***B^1-5^***) and to the ring-adjacent region (***F***, highlighted with yellow in ***B^1,2^***). Each series of three images (***F***) shows the pixel-based ΔF/F taken from the sample shown in ***B*** and averaged over the time course before (top left), during (top middle), or after (top right) stimulation. Bottom, The time course of fluorescence changes in similar regions of interest from six independent samples, binned into seconds, and sorted by the average fluorescence change during stimulation from most to least. For ***E***, ***F***, Extended Data 1 contains the complete data.

We created a fly that expressed CsChrimson specifically in Tk-GAL4^FruM^ neurons and the genetically encoded calcium indicator GCaMP6f specifically in *TkR86C^LexA^* neurons. We used two-photon serial volumetric imaging to monitor the fluorescence intensity of GCaMP6f in multiple *z*-planes (dorsal to ventral) of the brain in live flies (Siju et al., 2020) while Tk-GAL4^FruM^ cells were activated with an external LED (Fig. 3*A*). On LED stimulation, we observed localized increases in GCaMP6f fluorescence (Fig. 3*B*). The largest and most consistent change in fluorescence was observed in the *TkR86C^LexA^* neuronal processes that were near the SMP presynaptic sites of Tk-GAL4^FruM^ neurons (Fig. 3*C–E*). The activated domain extended posterior to the presynaptic area of Tk-GAL4^FruM^ neurons while remaining clearly compartmentalized. We did not observe such an increase in calcium activity in areas overlapping with ring-adjacent postsynaptic projections (Fig. 3*F*). Although we occasionally observed fluorescence fluctuations in other areas of the brain (Fig. 3*B2*), this was weaker and less consistent than the activity in the SMP.

**Figure 4.**
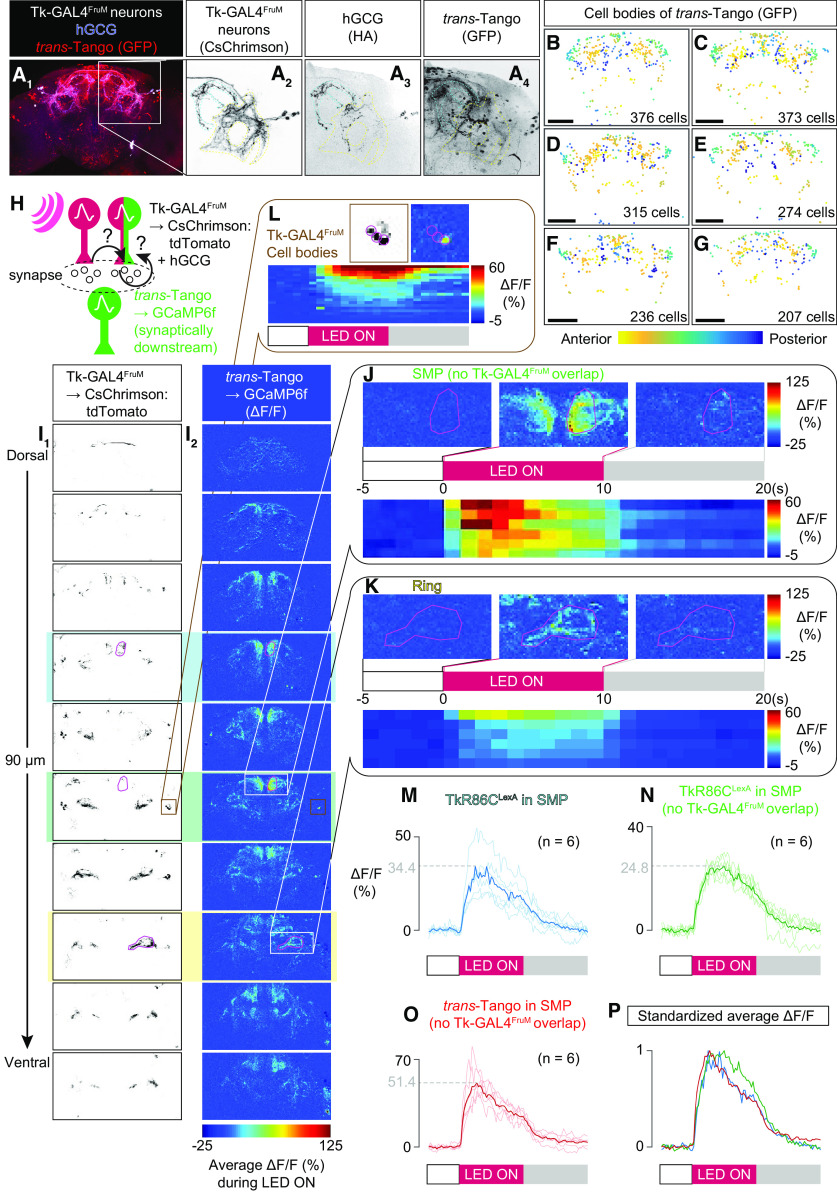
Synaptic downstream of Tk-GAL4^FruM^ neurons likely contains *TkR86C^LexA^* neurons. ***A_1–4_***, A representative image of a male brain expressing CsChrimson:tdTomato (***A_1_***, ***A_2_***, white) and membrane-tethered human glucagon (hGCG; ***A_1_***, ***A_3_***, blue), the ligand of *trans*-Tango, in Tk-GAL4^FruM^ neurons. GFP expressed by the *trans*-Tango transgene is overlaid (***A_1_***, ***A_4_***, red). ***B–G***, Location of cell bodies labeled by trans-Tango in Tk-GAL4^FruM^ neurons in six independent brain samples with color of dots indicating anterior–posterior position. ***H***, A schematic representation of functional imaging of synaptically downstream partners of Tk-GAL4^FruM^ neurons using *trans*-Tango. *trans*-Tango may label Tk-GAL4^FruM^ neurons either via lateral connections among the group of cells or via self-labeling because of the presence of the receptor component of *trans*-Tango in these cells. ***I_1_***, ***I_2_***, Images of CsChrimson:tdTomato fluorescence in Tk-GAL4^FruM^ neurons (***I_1_***) and average normalized change in GCaMP6f fluorescence (ΔF/F) in putative synaptic downstream partners of Tk-GAL4^FruM^ neurons labeled by *trans*-Tango (***I_2_***) in a representative sample during optogenetic stimulation of Tk-GAL4^FruM^ neurons. ***J***, ***K***, Pseudocolored GCaMP6f fluorescence changes (ΔF/F) from the area posterior to the projection of Tk-GAL4^FruM^ neurons to the SMP (***J***, highlighted with green in ***I***) or the ring-adjacent region (***K***, highlighted with yellow in ***I***), represented as a spatiotemporal average from the sample shown in ***I*** (top) and as a time course binned into seconds from six independent samples (bottom). Unlike neurons labeled by *TkR86C^LexA^* (Fig. 3), calcium increased in the area near the projection of Tk-GAL4^FruM^ neurons to the ring-adjacent region. ***L***, Top, Magnified images of CsChrimson:tdTomato fluorescence in Tk-GAL4^FruM^ neurons (left) and pseudocolored GCaMP6f fluorescence changes (ΔF/F) in cell bodies of Tk-GAL4^FruM^ neurons (right), from the optical slice shown in ***I***. Cell bodies are demarked in magenta. Note the increase in GCaMP6f fluorescence in the rightmost cell. Bottom, Time course of pseudocolored GCaMP6f fluorescence changes (ΔF/F), binned into seconds, from 21 cell bodies in 7 independent brains. For ***B***, ***D***, ***E***, Extended Data 1 contains the complete data. ***M***, ***N***, Fluorescence changes (ΔF/F) in *TkR86C^LexA^* neurons in the vicinity of (***M***) and in the area 10–20 µm posterior to (***N***) the Tk-GAL4^FruM^ neuronal projection in the SMP. ***O***, Fluorescence changes (ΔF/F) in *trans-*Tango neurons in the area 10–20 µm posterior of Tk-GAL4^FruM^ neuronal projections in the SMP. Thick colored lines represent the average of the samples tested (thin lines). ***P***, An overlay of the averages from ***L*** to ***N***, normalized to the maximum fluorescence increase. For ***J***, ***K***, ***L***, ***M***, ***N***, ***O***, ***P***, Extended Data 1 contains the complete data.

The fluorescence increase observed in the SMP began at the onset of LED stimulation and increased rapidly for ∼2 s before starting to gradually decline even during the LED pulses (Fig. 3*E*). The fluorescence dropped when the LED was turned off, returning to the baseline in a few seconds in most cases. These spatial and temporal dynamics suggest that calcium activity in *TkR86C^LexA^* neurons is largely correlated with the activation of Tk-GAL4^FruM^ neurons. Importantly, these temporal dynamics were closely recapitulated when genetically defined, synaptically downstream neurons were accessed via the *trans*-Tango approach (Talay et al., 2017). Membrane-tethered human glucagon (hGCG) expressed in Tk-GAL4^FruM^ neurons drove expression of GCaMP6f in 200 ∼ 400 candidate synaptically downstream neurons across the brain (Fig. 4*A–G*). We then monitored LED stimulation-dependent calcium changes in these synaptically downstream neurons in response to optogenetic activation of Tk-GAL4^FruM^ neurons (Fig. 4*H*). Reflecting the rather widespread distribution of postsynaptic neurons, the fluorescent calcium activity was more widespread in *trans*-Tango samples than in brains expressing GCaMP6f under *TkR86C^LexA^* (Fig. 4*I*) and included activity in the ring-adjacent regions (Fig. 4*K*). Part of the activity in the ring-adjacent area was generated by occasional GCaMP6f expression in Tk-GAL4^FruM^ neurons themselves (Fig. 4*L*), because of either lateral connectivity among Tk-GAL4^FruM^ neurons or self-labeling by *trans*-Tango. Nonetheless, we consistently observed a fluorescence increase in the region posterior to (but not overlapping) the SMP projections of Tk-GAL4^FruM^ neurons (Fig. 4*I*,*J*). The activation patterns observed in the SMP were spatially and temporarily similar to the fluorescence dynamics observed in *TkR86C^LexA^* neurons (Fig. 4*M–P*). Although we could not colabel *trans*-Tango neurons with *TkR86C^LexA^* because of the low eclosion rate of the desired genotype (likely a consequence of many transgenes), the functional imaging data support the notion that GCaMP6f signals in *TkR86C^LexA^* neurons result from direct postsynaptic connections with Tk-GAL4^FruM^ neurons.

### Cholinergic transmission is critical for the excitation of downstream *TkR86C^LexA^* neurons

**Figure 5.**
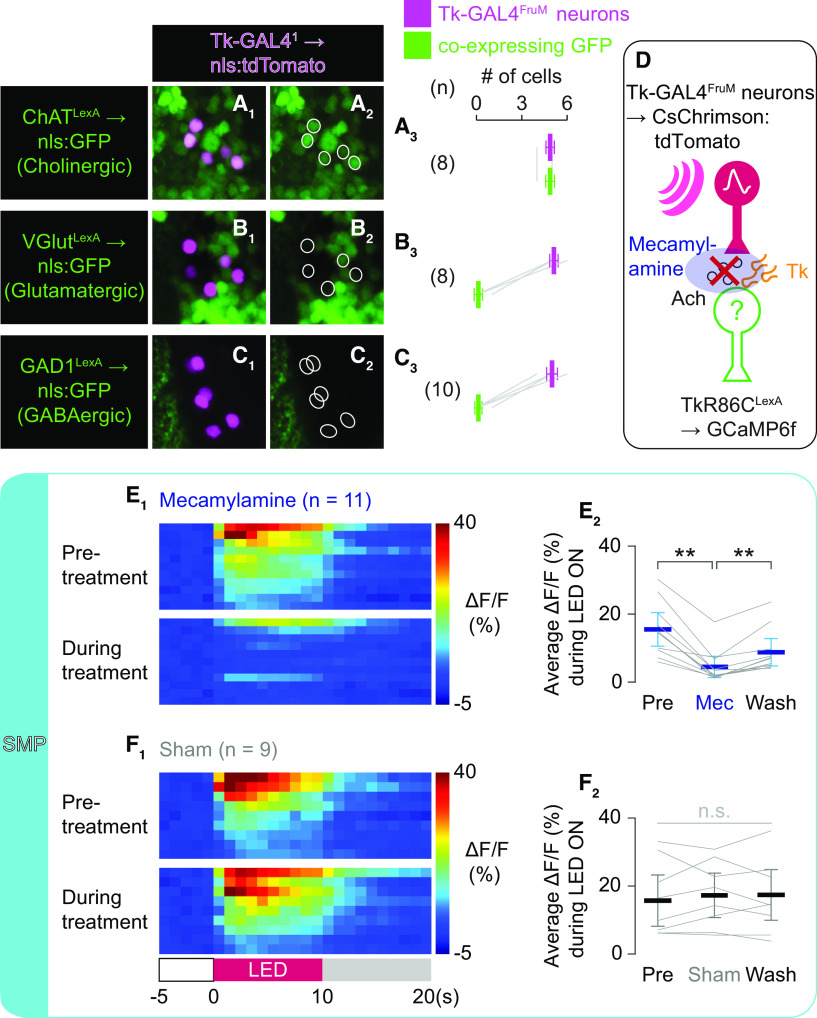
*TkR86C^LexA^* neurons receive cholinergic input from Tk-GAL4^FruM^ neurons. ***A_1_–C_3_***, Overlap of nuclear-localizing tdTomato in Tk-GAL4^FruM^ neurons (magenta) and nuclear-localizing GFP in cholinergic (***A***), glutamatergic (***B***), and GABAergic (***C***) neurons (green). ***D–F_2_***, A schematic representation of the functional imaging experiment for ***E*** and ***F*** in which mecamylamine blocks cholinergic neurotransmission. Fluorescence change (ΔF/F) from *TkR86C^LexA^* neurons in the vicinity of the Tk-GAL4^FruM^ neuronal projection in the SMP with mecamylamine application (***E***) or sham treatment (***F***). Pseudocolored ΔF/F (***E_1_***, ***F_1_***) represents fluorescence time courses for individual brains binned into seconds before (top) and during (bottom) treatment. Brain time courses are sorted by the average fluorescence change during stimulation, from most to least. The average ΔF/F from the same sample set before, during, and after treatment (***E_2_***, ***F_2_***) is shown on the right; **p < 0.01 by repeated-measures ANOVA and *post hoc* paired *t* test; n.s. (in gray) *p* > 0.05 by repeated-measures ANOVA. Thick lines and error bars in ***A_3_***–***C_3_***, ***E_2_***, and ***F_2_*** represent the average and 95% confidence intervals. For ***A***, ***B***, ***C***, ***E***, ***F***, Table 9 and Extended Data 1 contain the complete data and statistical results.

The increase in intracellular calcium concentration in *TkR86C^LexA^* neurons with Tk-GAL4^FruM^ stimulation suggests that the overall impact of Tk-GAL4^FruM^ neuronal transmission is excitatory. Consistent with this and with previous observations (Asahina et al., 2014), we found evidence that Tk-GAL4^FruM^ neurons coexpress *choline acetyltransferase* (*ChAT*), a marker for excitatory cholinergic neurons (Fig. 5*A*), but not markers for glutamatergic (Fig. 5*B*) or GABAergic (Fig. 5*C*) neurons. Peptidergic ligands of *TkR86C* increase intracellular calcium concentration (Poels et al., 2009; Jiang et al., 2013), suggesting that the GCaMP6f signals we observed from *TkR86C^LexA^* neurons are a combination of cholinergic and tachykininergic transmission. To parse out the contribution of each of the two transmitter types, we first blocked cholinergic signaling with mecamylamine, an antagonist of the nicotinic acetylcholine receptor (Fig. 5*D*). The increase in GCaMP6f fluorescence in *TkR86C^LexA^* neurons triggered by optogenetic stimulation of Tk-GAL4^FruM^ neurons was severely reduced after bath application of mecamylamine and could be partially rescued with a wash out (Fig. 5*E1*,*2*). By contrast, calcium signals remained largely unchanged when vehicle was added to the bath (Fig. 5*F1*,*2*). These data suggest that cholinergic signaling is a major contributor to the calcium activity observed in *TkR86C^LexA^* neurons on Tk-GAL4^FruM^ neuronal activation.

**Figure 6.**
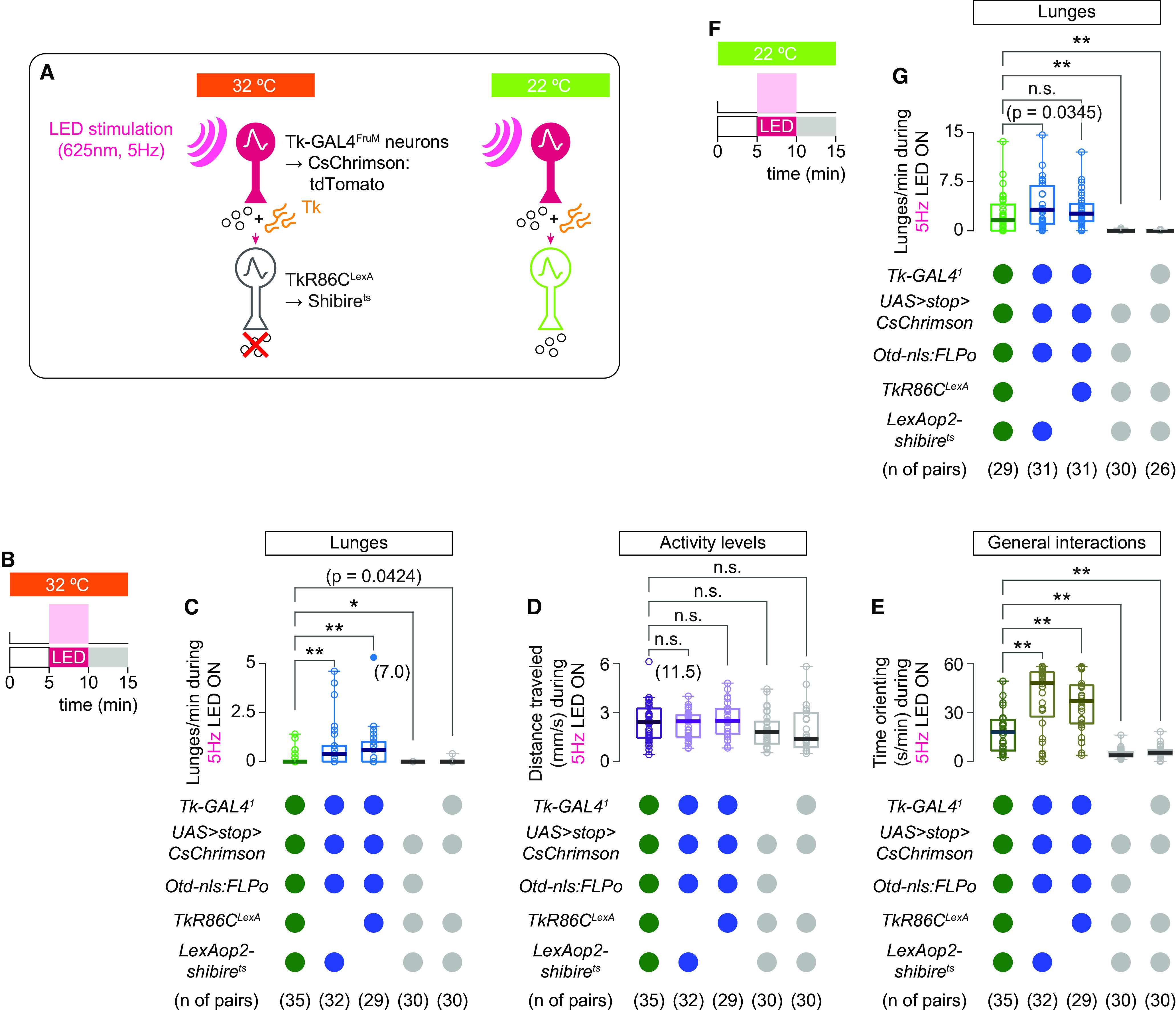
Blocking of synaptic transmission from *TkR86C^LexA^* neurons suppresses aggression induced by optogenetic activation of Tk-GAL4^FruM^ neurons. ***A***, Schematic of the genetic manipulations used in the behavioral experiment in this figure. ***B***, Design of the optogenetic behavioral experiments in the restrictive temperature of 32°C. ***C***, Box plot of number of lunges performed by the flies with the indicated genotypes during each time window; ** *p* < 0.01, * *p* < 0.05 by Kruskal–Wallis one-way ANOVA and *post hoc* Mann–Whitney *U* test. ***D***, Box plots of distance traveled by the same pairs of flies as in ***C***; n.s. *p* > 0.05 by Kruskal–Wallis one-way ANOVA and *post hoc* Mann–Whitney *U* test. ***E***, Box plots of duration of orienting toward an opponent (the same pairs of flies as in ***C***); ** *p* < 0.01 by Kruskal–Wallis one-way ANOVA and *post hoc* Mann–Whitney *U* test. ***F***, Design of the optogenetic behavioral experiments in the permissive temperature of 22°C. ***G***, Box plot of number of lunges performed by the flies with the indicated genotypes during each time window; ** *p* < 0.01 by Kruskal–Wallis one-way ANOVA and *post hoc* Mann–Whitney *U* test. For ***C***, ***D***, ***E***, ***G***, Table 9 and Extended Data 1 contain the complete data and statistical results.

We reasoned that blocking synaptic transmission from *TkR86C^LexA^* neurons should prevent Tk-GAL4^FruM^ neurons from promoting aggression if these neurons are the major recipient of synaptic output from Tk-GAL4^FruM^ neurons. To test this possibility, we optogenetically activated Tk-GAL4^FruM^ neurons while blocking neurotransmission from *TkR86C^LexA^* neurons with the temperature-sensitive mutant protein of dynamin, Shibire^ts^ (Kitamoto, 2001; Fig. 6*A*). At a restrictive temperature of 32°C, where Shibire^ts^ is expected to block neurotransmission of *TkR86C^LexA^* neurons, optogenetic stimulation of Tk-GAL4^FruM^ neurons induced significantly fewer lunges in the mutant than in genetic controls (Fig. 6*B*,*C*). In contrast, at the permissive temperature of 22°C, the number of lunges during LED stimulation was comparable between the experimental and control genotypes (Fig. 6*F*,*G*), indicating that neurotransmission from *TkR86C^LexA^* neurons is necessary for Tk-GAL4^FruM^ neurons to promote aggression. Because *TkR86^LexA^* neurons are numerous in the nervous system, including in the ventral nerve cord (Fig. 2*M*), we cannot completely rule out a role for *TkR86^LexA^* neurons in general motor function. However, distance traveled during LED stimulation was comparable in experimental and control genotypes (Fig. 6*D*). Duration of orienting toward a target fly, a proxy of general interactions (Wohl et al., 2020), in the experimental genotype was decreased compared with the two control genotypes that did not express Shibire^ts^ in *TkR86C^LexA^* neurons (Fig. 6*E*) but was increased compared with the two control genotypes that did not express CsChrimson in Tk-GAL4^FruM^ neurons. This result indicates that the expression of Shibire^ts^ proteins did not prevent flies from interacting. These data collectively suggest that blocking *TkR86C^LexA^* neuronal transmission does not impair basic motor function. We conclude that *TkR86C*-expressing neurons receive cholinergic synaptic inputs from Tk-GAL4^FruM^ neurons and are necessary for Tk-GAL4^FruM^ neurons-induced aggression.

### Tachykinin modulates excitatory postsynaptic responses in *TkR86C^LexA^* neurons

**Figure 7.**
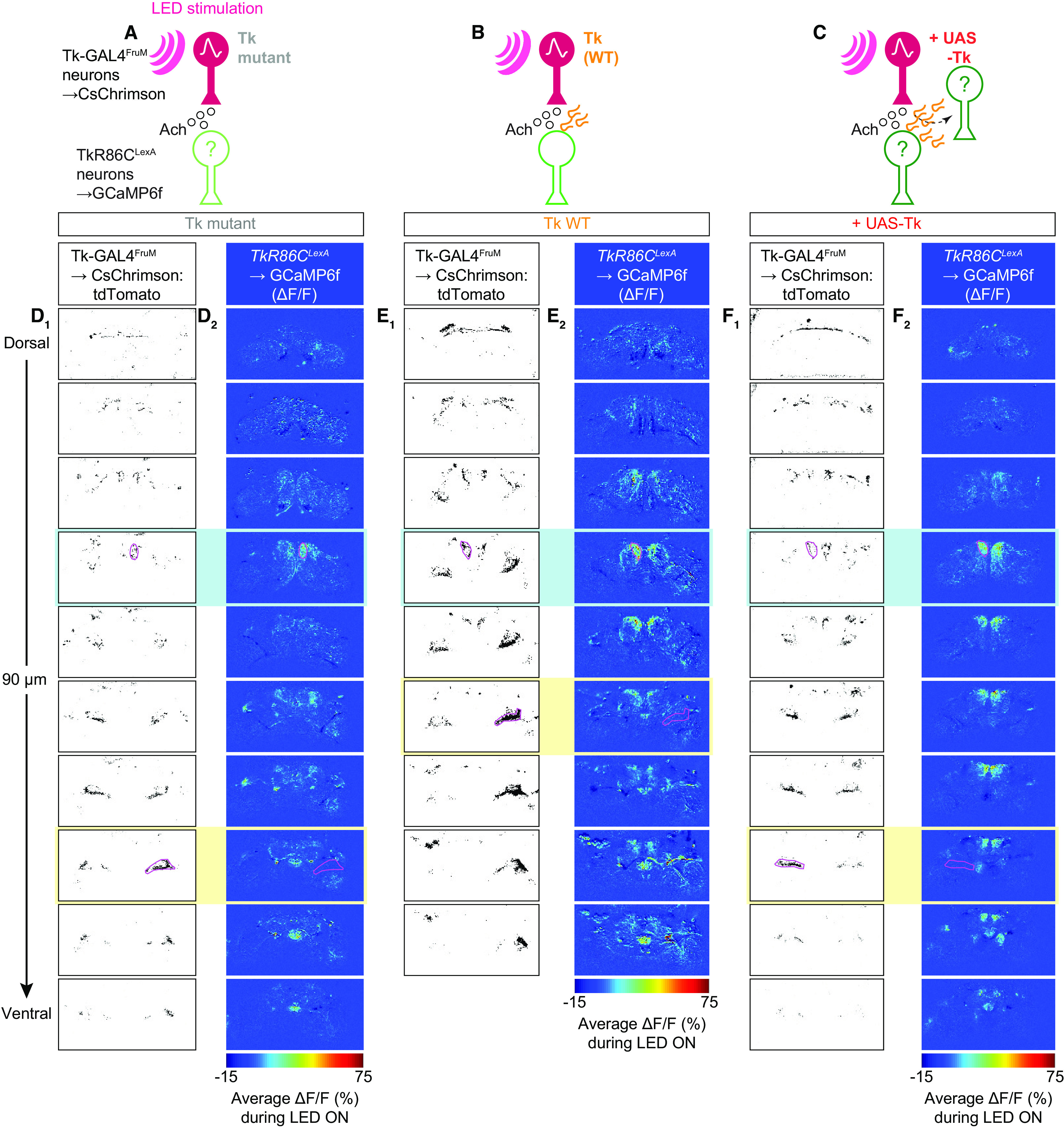
Responses of *TkR86C^LexA^* neurons by the optogenetic activation Tk-GAL4^FruM^ neurons with different amount of tachykinin. ***A–F_2_***, Schematic representation of functional imaging experiments from *TkR86C^LexA^* neurons in *Tk* null mutants (***A***, data in ***D***), *Tk* wild type (***B***, data in ***E***), and *Tk* overexpression with a *UAS-Tk* transgene (***C***, data in ***F***). Images of CsChrimson:tdTomato fluorescence in Tk-GAL4^FruM^ neurons (***D_1_***, ***F_1_***) and average fluorescence of GCaMP6f in *TkR86C^LexA^* neurons (***D_2_***, ***F_2_***) in a representative sample from *Tk* null mutants (***D***), *Tk* wild type (***E***), and in an animal with a *UAS-Tk* transgene (***F***). The GCaMP6f signals are shown in pseudocolor (scales at the bottom of ***D_2_***, ***E_2_***, and ***F_2_***) as the relative increase of fluorescence (ΔF/F) during optogenetic activation of Tk-GAL4^FruM^ neurons. Frames with cyan and yellow backgrounds were used for measurements of the responses in the SMP and the ring-adjacent region, respectively (Fig. 8).

How does tachykinin modulate the cholinergic excitatory input from Tk-GAL4^FruM^ neurons onto *TkR86C^LexA^* neurons? To answer this question, we quantified the excitatory responses of *TkR86C^LexA^* neurons to optogenetic excitation of Tk-GAL4^FruM^ neurons while either eliminating (Fig. 7*A*,*B*) or overexpressing (Fig. 7*C*) *Tk*. As shown in Figure 1, manipulating the amount of *Tk* changes how strongly Tk-GAL4^FruM^ neurons promote aggression on optogenetic activation.

**Figure 8.**
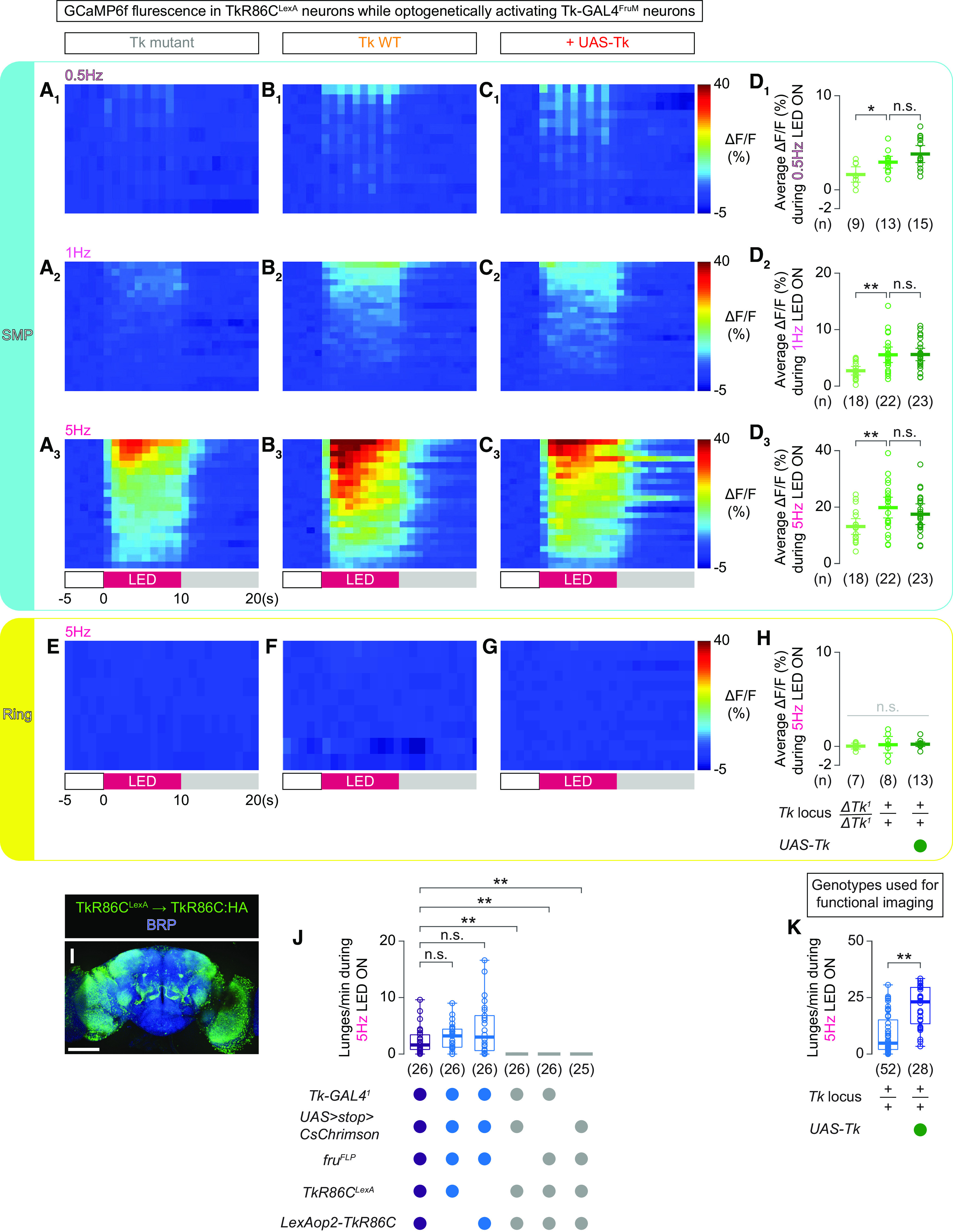
Tachykinin is necessary to maintain the wild-type intensity of excitatory synaptic transmission from Tk-GAL4^FruM^ neurons to *TkR86C^LexA^* neurons. ***A_1_–H***, Pseudocolored time course binned into seconds of GCaMP6f fluorescence changes (ΔF/F) in *TkR86C^LexA^* neurons, sorted by the average fluorescence change during stimulation, from the vicinity of the Tk-GAL4^FruM^ neuronal projection in the SMP (***A–C***) or in the ring-adjacent region (***E–G***). The SMP was imaged at three different LED frequencies as indicated above (***A_1-3_***). Average ΔF/F in *TkR86C^LexA^* neurons during optogenetic stimulation of Tk-GAL4^FruM^ neurons from the vicinity of the Tk-GAL4^FruM^ neuronal projection in the SMP (***D***) or in the ring-adjacent region (***H***); **p < 0.01, *p < 0.05 by one-way ANOVA and *post hoc t* test; n.s. (***H***, gray) *p* > 0.05 by one-way ANOVA. Thick lines and error bars in ***D_1-3_*** and ***H*** represent the average and 95% confidence intervals. Data points (***D***, ***H***, left) are from *Tk* null mutants (light green), the middle data points are from *Tk* wild type (green), and the right data points are from animals with a *UAS-Tk* transgene (dark green), with *n* indicated at the bottom. ***I***, Representative expression pattern of TkR86C tagged with HA (green) driven by *TkR86C^LexA^* in the brain along with the neuropil marker Brp, visualized by immunohistochemistry. Scale bar, 100 µm. ***J***, Box plots of lunges performed during optogenetic stimulation of Tk-GAL4^FruM^ neurons while TkR86C is overexpressed by *TkR86C^LexA^* (purple, right), along with genetic controls as indicated (bottom); **p < 0.01, n.s. *p* > 0.5 by Kruskal–Wallis one-way ANOVA and *post hoc* Mann–Whitney *U* test. ***K***, Box plots of lunges performed during optogenetic stimulation of Tk-GAL4^FruM^ neurons, with (right) or without (left) the *UAS-Tk* transgene, by the genotypes used for the functional imaging. For ***A–H*, *J***, ***K***, Table 9 and Extended Data 1 contain the complete data and statistical results.

In *Tk* null mutants, the increase in GCaMP6f fluorescence in the SMP evoked by optogenetic stimulation of Tk-GAL4^FruM^ neurons was significantly attenuated compared with animals with the wild-type *Tk* locus at multiple stimulation frequencies (Figs. 7*D*,*E*, 8*A*,*B*,*D*). The average increase in fluorescence (ΔF/F) was 30–50% lower in the *Tk* mutants than in wild type, which parallels the reduction in lunges induced by optogenetic stimulation of Tk-GAL4^FruM^ neurons under comparable LED power and frequencies (Fig. 1*B*,*C*,*E–G*,*I*). These data suggest that tachykinin is necessary for maintaining the strength of excitatory transmissions between Tk-GAL4^FruM^ neurons and downstream *TkR86C^LexA^* neurons. The presence of responses in *TkR86C^LexA^* neurons in the *Tk* null background, albeit reduced, also suggests that acetylcholine alone can sustain some functional connectivity in the absence of tachykinin, reflecting the reduction but not elimination of aggression induced by Tk-GAL4^FruM^ excitation in *Tk* null mutants (Fig. 1*B*,*F*; Asahina et al., 2014).

Interestingly, overexpression of tachykinin in Tk-GAL4^FruM^ neurons did not further increase GCaMP6f fluorescence in the SMP compared with the signals in animals with a wild-type *Tk* locus (Figs. 7*E*,*F*, 8*B–D*), although the same genetic manipulation induced more lunges when Tk-GAL4^FruM^ neurons were activated at the same LED power and frequency (Fig. 1*C–E*,*G–I*). We did not observe any gross spatial changes in GCaMP6f signals from *TkR86C^LexA^* neurons when the *Tk* amounts were manipulated (Fig. 7*E*,*F*), including arbors near the ring-adjacent region of Tk-GAL4^FruM^ neurons (Fig. 8*E–H*). The absence of a difference in the response magnitude in the SMP may be because of the saturation of receptors in *TkR86C^LexA^* neurons. In fact, the level of receptor expression limits the efficacy of tachykininergic neuromodulation in olfactory and nociceptive circuits (Ignell et al., 2009; Im et al., 2015; Ko et al., 2015). However, overexpression of TkR86C in *TkR86C^LexA^* neurons did not further enhance aggression induced by the optogenetic activation of Tk-GAL4^FruM^ neurons (Fig. 8*I*,*J*). This suggests that the amount of tachykinin, rather than TkR86C receptors, is the limiting factor for the level of aggression. Moreover, optogenetic stimulation of Tk-GAL4^FruM^ neurons induced more lunges with *Tk* overexpression when the neurons also expressed GCaMP6f (Fig. 8*K*), excluding the possibility that GCaMP6f interferes with the aggression-promoting impact of *Tk* overexpression. These data collectively support the conclusion that excess tachykinin in *Tk-GAL4^1^* neurons does not change the dynamics of the circuit that involves *TkR86C^LexA^* neurons, although it both quantitatively and qualitatively enhances aggression induced by the optogenetic activation of Tk-GAL4^FruM^ neurons.

### Tachykinin overexpression in Tk-GAL4^FruM^ neurons recruits *TkR99D*-expressing neurons

**Figure 9.**
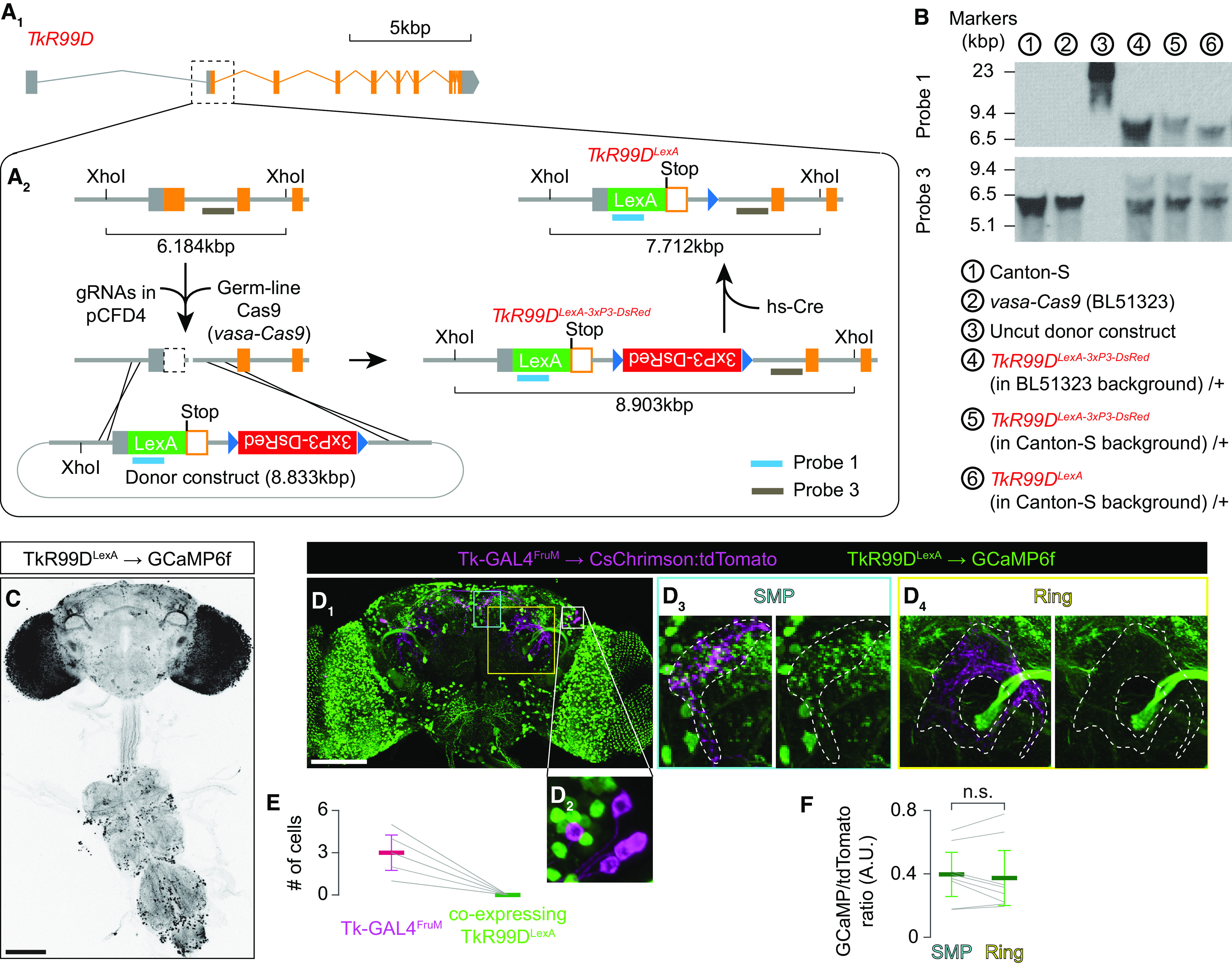
Characterization of the *TkR99D^LexA^* allele and neurons visualized by the allele. ***A_1_***, ***A_2_***, A schematic of the steps taken to create the *TkR99D^LexA^* allele. The second exon of *TkR99D* was modified (***A_1_***) with CRISPR/Cas9-mediated homologous recombination as described in ***A_2_***. ***B***, Southern blotting analysis of *TkR99D^LexA^* alleles. Probe 1 targets the coding region of LexA (also used in Fig. 2*K2*, 2*L*) whereas probe 3 targets the area downstream of the *TkR99D* second exon (***A_2_***). Note that flies 4–6 were heterozygous for the *TkR99D* alleles, therefore ∼6.2 kb fragments from the wild-type allele were also present. ***C***, Representative expression pattern of GCaMP6f driven by *TkR99D^LexA^* in the nervous system, visualized by immunohistochemistry. Scale bar, 100 µm. ***D_1–4_*_,_** Representative image of a male brain expressing GCaMP6f driven by *TkR99D^LexA^* (green) and CsChrimson:tdTomato under intersectional control of *Tk-GAL4^1^* and *fru^FLP^* (magenta; ***D_1_***). Images near the Tk-GAL4^FruM^ cell bodies (***D_2_***) and their projections (dashed white lines) in the SMP (***D_3_***) and the ring (***D_4_***) regions are magnified. Scale bar, 100 µm. ***E***, *TkR99D^LexA^* does not label Tk-GAL4^FruM^ neurons (*n* = 6 hemibrains from 3 brains). ***F***, Box plot of GCaMP6f immunohistochemical signals in the SMP and ring-adjacent regions defined by Tk-GAL4^FruM^ neurons, relative to their tdTomato immunohistochemical signals (*n* = 8 hemibrains from 4 brains); n.s. *p* > 0.05 by paired *t* test. For ***E***, ***F***, Table 9 and Extended Data 1 contain the complete data and statistical results.

The absence of a noticeable difference in *TkR86C^LexA^* calcium signals with tachykinin overexpression suggests that these are not the only neural correlates of enhanced aggression induced by activation of Tk-GAL4^FruM^ neurons. We asked whether another tachykinin receptor, *TkR99D* (Birse et al., 2006), plays a role in defining a parallel behaviorally relevant circuit. Although not required for aggression induced by the activation of Tk-GAL4^FruM^ neurons (Asahina et al., 2014), TkR99D receptor proteins may detect overexpressed tachykinin from Tk-GAL4^FruM^ neurons (which can increase the local concentration of tachykinin), perhaps without direct synaptic connection, given the higher affinity of this receptor to tachykinin than TkR86C (Birse et al., 2006; Poels et al., 2009; Jiang et al., 2013). To address this possibility, we created a LexA knock-in allele of *TkR99D* with the same strategy used for *TkR86C^LexA^* (Fig. 9*A*,*B*). Like *TkR86C^LexA^*, *TkR99D^LexA^* labeled many neurons throughout the brain (Fig. 9*C*), but not Tk-GAL4^FruM^ neurons themselves (Fig. 9*D1*,*2*,*E*). In contrast to *TkR86C^LexA^* neurons, the overlap of *TkR99D^LexA^* neurons near the presynaptic projections of Tk-GAL4^FruM^ neurons in the SMP was comparable to that in the postsynaptic regions (Fig. 9*D3*,*4*,*F*).

**Figure 10.**
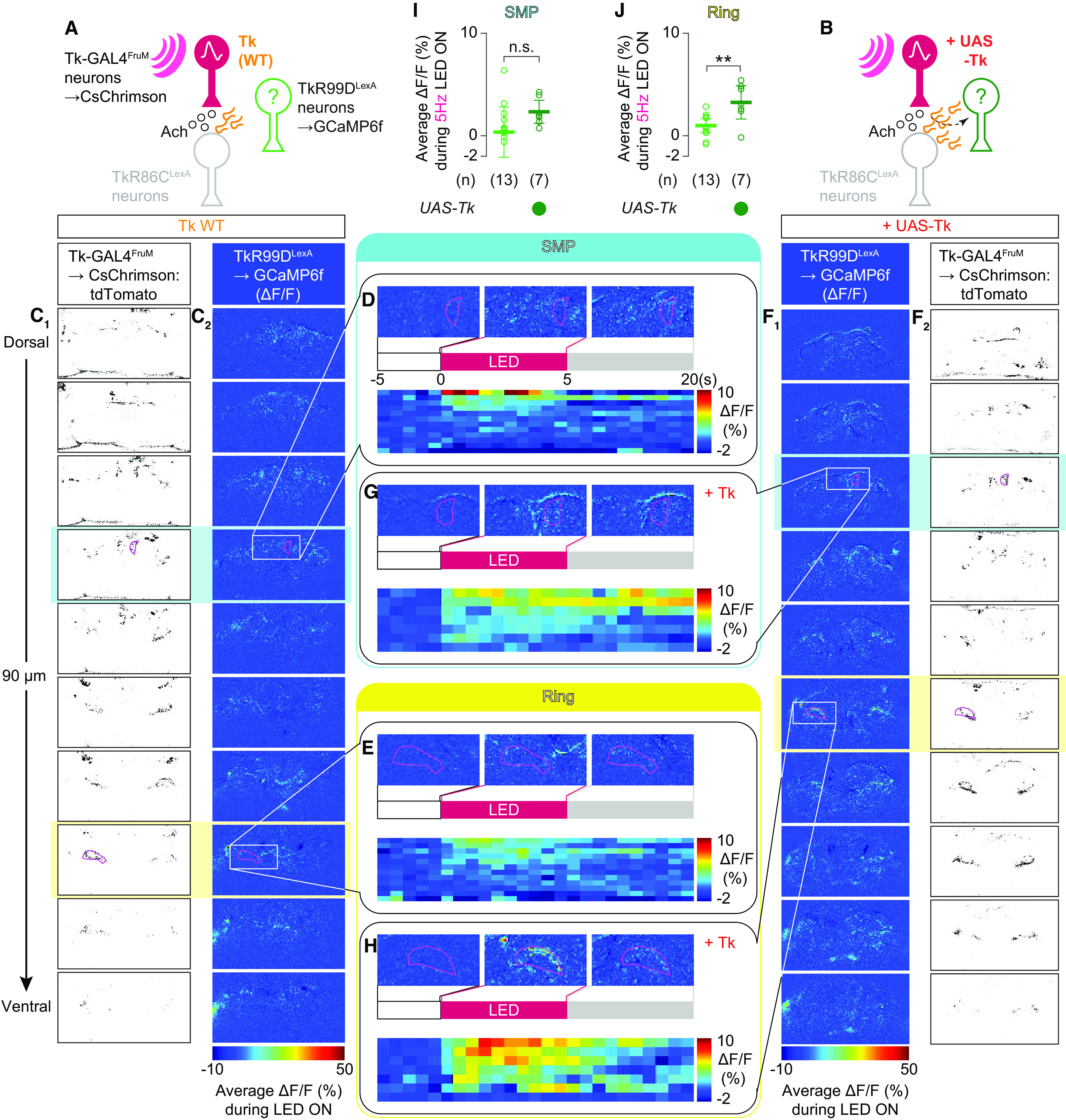
*TkR99D^LexA^* neurons are consistently activated by Tk-GAL4^FruM^ neurons when tachykinins are overexpressed. ***A***, ***B***, Schematic representations of functional imaging experiments from *TkR99D^LexA^* neurons in *Tk* wild type (***A***) and in the presence of a *UAS-Tk* transgene (***B***). ***C_1_–H***, Images of CsChrimson:tdTomato fluorescence in Tk-GAL4^FruM^ neurons (***C_1_***, ***F_2_***) and average fluorescence of GCaMP6f in *TkR99D^LexA^* neurons (***C_2_***, ***F_1_***) in a representative sample from a *Tk* wild-type animal (***C***), and in an animal with a *UAS-Tk* transgene (***F***). The GCaMP6f signals are shown in pseudocolor (scales at the bottom of C2, F2) as the relative increase in fluorescence (ΔF/*F*) during optogenetic activation of Tk-GAL4^FruM^ neurons. ***D***, ***E***, ***G***, ***H***, Pseudocolored GCaMP6f fluorescence changes (ΔF/F) in the vicinity of the projections of Tk-GAL4^FruM^ neurons to the SMP (***D***, ***G***, from frames with cyan background in ***C***, ***F***) and to the ring-adjacent region (***E***, ***H***), in *Tk* wild type (***D***, ***E***, from frames with yellow background in ***C***, ***F***) and in the presence of a *UAS-Tk* transgene (***G***, ***H***). Each series of three images shows the pixel-based ΔF/F taken from the sample shown in ***C*** (for ***D***, ***E***) or ***F*** (for ***G***, ***H***) and averaged over the time course before (left), during (middle), or after (right) stimulation. Bottom, The time course of fluorescence changes in similar regions of interest from independent samples (*n* = 13, ***D***, ***E***; *n* = 7, ***G***, ***H***), binned into seconds and sorted by the average fluorescence change during stimulation, from most to least. ***I***, ***J***, Average ΔF/F in *TkR99D^LexA^* neurons during optogenetic stimulation of Tk-GAL4^FruM^ neurons, from the vicinity of the Tk-GAL4^FruM^ neuronal projection in the SMP (***I***) or in the ring-adjacent region (***J***). Left, data points (***I***, ***J***) are from *Tk* wild-type and data points (right) are from animals with a *UAS-Tk* transgene. Bottom, *n* is indicated; **p < 0.01, n.s. *p* > 0.05 by *t* test. For ***D***, ***E***, ***G***, ***H***, ***I***, ***J***, Table 9 and Extended Data 1 contain the complete data and statistical results.

We next asked whether any *TkR99D^LexA^* neurons are functionally downstream of Tk-GAL4^FruM^ neurons. We expressed GCaMP6f under the control of *TkR99D^LexA^* while expressing CsChrimson in Tk-GAL4^FruM^ neurons (Fig. 10*A*) and monitored fluorescence intensity in response to optogenetic stimulation of Tk-GAL4^FruM^ neurons. We did not observe consistent fluorescence fluctuations near the innervation from Tk-GAL4^FruM^ neurons, either in the SMP (Fig. 10*B*,*C*) or in the ring-adjacent region (Fig. 10*B*,*D*). We noticed that GCaMP6f intensity often increased after LED stimulation in the protocerebral bridge (Fig. 10*B*), where Tk-GAL4^FruM^ neurons do not project. This neural structure is known to respond to visual stimuli in both *Drosophila* (Weir and Dickinson, 2015) and other insect species (Heinze and Homberg, 2007; Homberg et al., 2011; Phillips-Portillo, 2012; Pegel et al., 2019). Therefore, direct activation of this visual circuit by the LED light may have led to the observed calcium response.

Interestingly, when *Tk* was overexpressed in Tk-GAL4^FruM^ neurons (Fig. 10*E*), optogenetic activation elevated GCaMP6f fluorescence near Tk-GAL4^FruM^ neurons (Fig. 10*F*,*G*,*H*). The fluorescence increase near the ring-adjacent region of Tk-GAL4^FruM^ neurons was significantly higher than in animals with wild-type *Tk* loci (Fig. 10*H*,*J*), whereas the signal in the SMP remained comparable (Fig. 10*G*,*I*). These newly recruited *TkR99D^LexA^* neurons are distinct from the *TkR86C^LexA^* neurons that are synaptically downstream of Tk-GAL4^FruM^ neurons as *TkR86C^LexA^* neurons near the ring-adjacent region are not recruited by optogenetic activation of Tk-GAL4^FruM^ neurons that overexpress *Tk*. This suggests that tachykinins released from Tk-GAL4^FruM^ neurons can modulate two distinct circuits depending on the available amount of *Tk*.

**Figure 11.**
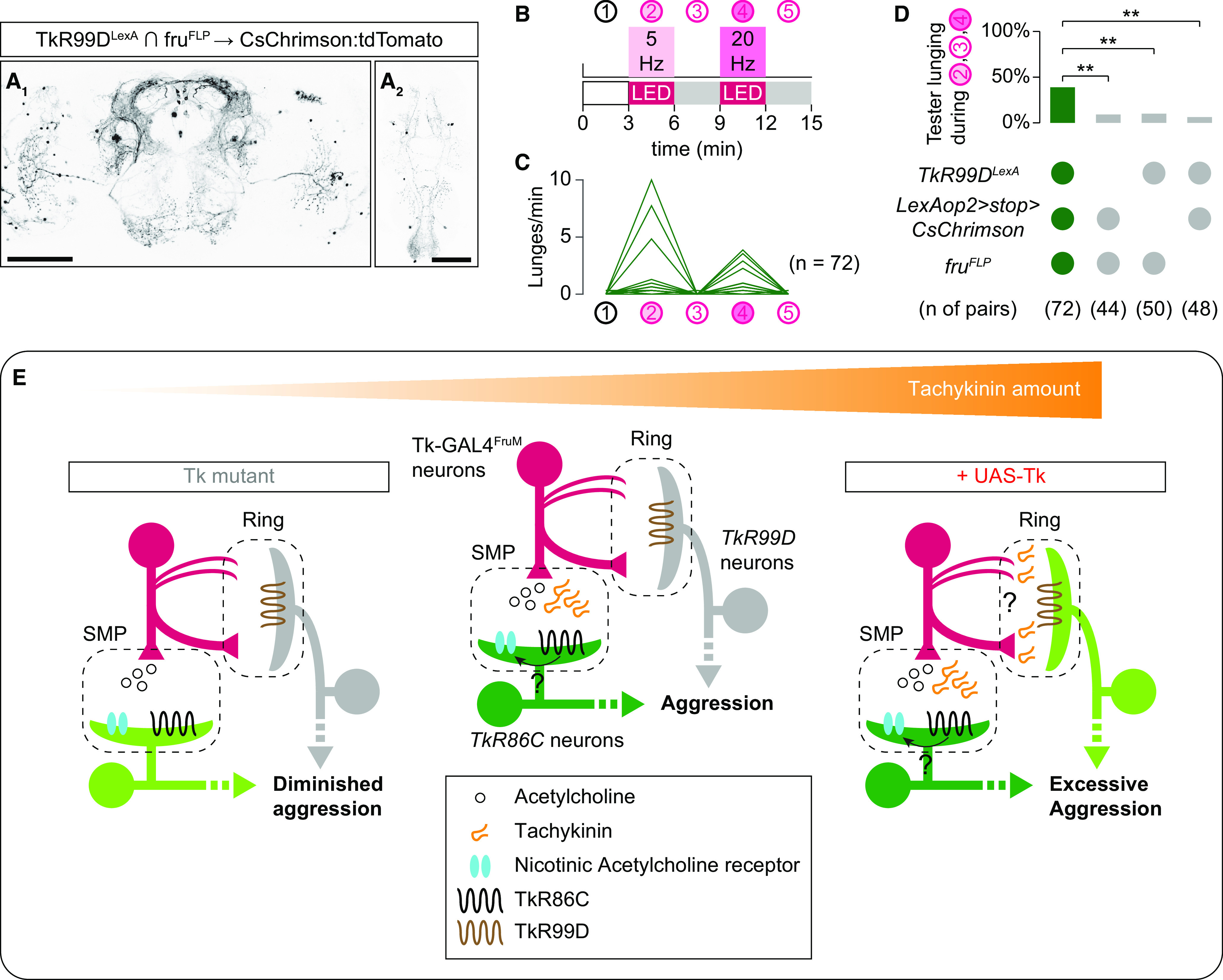
*TkR99D^LexA^* neurons influence aggression. ***A_1_***_,_***_2_***, Representative expression pattern of CsChrimson:tdTomato driven by the fru^FLP^-expressing subset of *TkR99D^LexA^* in the central brain (***A_1_***) and in the ventral nerve cord (***A_2_***), visualized by immunohistochemistry. Scale bar, 100 µm. ***B***, Design of the optogenetic behavioral assay. ***C***, The number of lunges by a fly with CsChrimson in neurons that express both *TkR99D^LexA^* and fru^FLP^ (***A***) toward a wild-type male fly, in each of the 5 time windows shown in ***B***. ***D***, The percentage of flies with the indicated genotypes that lunged from the onset of the first LED stimulation till the end of the second LED stimulation (***B***); **p < 0.01 by Fisher's exact test. For ***C***, ***D***, Table 9 and Extended Data 1 contain the complete data and statistical results. ***E***, A model of tachykininergic neuromodulation by Tk-GAL4^FruM^ neurons to two distinct downstream neuronal populations. Middle, Tk-GAL4^FruM^ neurons make cholinergic synaptic connections in the SMP with *TkR86C*-expressing downstream neurons, which mediate the aggression promoted by Tk-GAL4^FruM^ neurons. Tachykinin potentiates the excitatory transmission through an unknown mechanism. Left, In the absence of tachykinin, the *TkR86C*-expressing downstream population is not as effectively excited by Tk-GAL4^FruM^ neurons, resulting in a diminished level of aggression. Overexpression of tachykinin in Tk-GAL4^FruM^ neurons recruits *TkR99D*-expressing neurons that project to the ring-adjacent region. Although Tk-GAL4^FruM^ neurons send mainly postsynaptic arbors to the ring area, axon termini from the contralateral side also reach there. Tachykinin from either structure excites *TkR99D*-expressing neurons, which can contribute to excessive aggression.

Do *TkR99D^LexA^* neurons contain aggression-promoting subtypes? We found that a subset of *TkR99D^LexA^* neurons that also express *fruitless* (Fig. 11*A*) mildly induced lunges when optogenetically activated (Fig. 11*A–D*). The weak phenotype is consistent with a hypothesis that *TkR99D*-expressing neurons modulate aggression only when the *TkR86C-*expressing neurons that are synaptically downstream of Tk-GAL4^FruM^ neurons are already active (Fig. 11*E*). Our data support the idea that *Tk* overexpression in Tk-GAL4^FruM^ neurons potentiates their aggression-promoting capability by recruiting an additional population of neurons that receive tachykinin via *TkR99D*.

## Discussion

Although neuropeptides modulate a wide range of behaviors, the cellular and genetic basis of this modulation has remained elusive. Using functional imaging, we found that tachykinin released from Tk-GAL4^FruM^ neurons modulates two distinct circuits (Fig. 11*E*). One is likely a direct postsynaptic target that expresses *TkR86C*. These neurons are necessary for Tk-GAL4^FruM^ neurons to promote aggression, with tachykinin modulating the excitatory response triggered by the cotransmitter acetylcholine. The other circuit is labeled by *TkR99D*. These neurons were recruited specifically when Tk-GAL4^FruM^ neurons with a high level of tachykinin were activated, which may account for both the qualitative and quantitative enhancement of aggressive behaviors when tachykinin is overexpressed in Tk-GAL4^FruM^ neurons. Our results predict a mechanism by which neuropeptides engage multiple neural circuits labeled by distinct neuropeptide receptors to control behavior intensity.

A single neuropeptide species is often recognized by multiple receptors (Nässel and Winther, 2010; Griebel and Holsboer, 2012). Different receptors are often expressed in separate neuronal populations, suggesting that they delineate neural circuits that are distinct from one another. Although we are currently unable to visualize the overlap of *TkR86C^LexA^* neurons and *TkR99D^LexA^* neurons directly, we predict that the *TkR86C^LexA^* neurons and *TkR99D^LexA^* neurons that are activated by Tk-GAL4^FruM^ neurons are nonoverlapping populations for two reasons. First, they are spatially segregated. Optogenetic activation of Tk-GAL4^FruM^ neurons excites *TkR86C^LexA^* neurons located almost exclusively near the axon termini of Tk-GAL4^FruM^ neurons in the SMP, whereas the same manipulation excites *TkR99D^LexA^* neurons that have processes near the dendritic arbors of Tk-GAL4^FruM^ neurons in the ring-adjacent region. Second, *TkR86C^LexA^* neurons can be excited by optogenetic activation of Tk-GAL4^FruM^ neurons even in the absence of tachykinin peptides, whereas *TkR99D^LexA^* neurons are reliably excited only when *Tk* is overexpressed in Tk-GAL4^FruM^ neurons. A division of labor between *TkR86C* and *TkR99D* was also reported in the *Drosophila* internal sugar-sensing neurons (Musso et al., 2021) and in the metabolic modulation of locomotion (Lee et al., 2021).

On the basis of our findings, we propose a model in which neuropeptides from a single population of neurons sculpt the activity in two separate downstream targets defined by different receptors (Fig. 11*E*). Importantly, whether each receptor-expressing population downstream of Tk-GAL4^FruM^ contributes to specific aspects of escalation remains an unanswered question. Despite our multiple attempts, identification of specific subsets of receptor-expressing neurons that are recruited by Tk-GAL4^FruM^ neurons has been unsuccessful (data not shown). Labeling with photo-activatable GFP (Datta et al., 2008; Ruta et al., 2010; Aso et al., 2014) suffered from an inability to migrate to cell bodies, whereas *trans*-Tango expressed in Tk-GAL4^FruM^ neurons labeled hundreds of cells across the brain with intermingled neuronal processes, preventing us from characterizing the neuroanatomy with cellular resolution. Finally, electron-microscopy (EM)-based wiring diagrams can be visualized only for the female fly brain (Zheng et al., 2018; Scheffer et al., 2020), preventing us from tracing the downstream synaptic connections of male-specific neurons (such as Tk-GAL4^FruM^ neurons) using the EM volume.

A unique feature of peptidergic neuromodulation is the diversity of neuronal targets (Nässel, 2009; van den Pol, 2012; Nusbaum et al., 2017). Our brainwide functional imaging revealed restricted activity patterns in response to optogenetic stimulation of Tk-GAL4^FruM^ neurons, suggesting tachykinins in this context mainly act locally. The absence of *TkR86C^LexA^* or *TkR99D^LexA^* expression in Tk-GAL4^FruM^ neurons excludes autoaxonal or axoaxonal modulation of Tk-GAL4^FruM^ neurons. The spatiotemporal similarity of the activity patterns in genetically identified postsynaptic neurons and *TkR86C^LexA^* neurons suggests that *TkR86C* mediates postsynaptic enhancement of cholinergic neurotransmission. The fact that an acetylcholine receptor antagonist almost completely blocks the Tk-GAL4^FruM^ neuron-induced activity in *TkR86C^LexA^* neurons further supports this conclusion. The relationship between *TkR99D^LexA^* neurons and Tk-GAL4^FruM^ neurons remains unclear. Because *TkR99D^LexA^* neurons are activated in proximity to the dendritic areas of Tk-GAL4^FruM^ neurons, it is possible that they receive tachykinin released from the dendrites of Tk-GAL4^FruM^ neurons. On the other hand, ring-adjacent postsynaptic neurons that express *TkR99D* may be activated by the contralateral projection of Tk-GAL4^FruM^ neurons. Identification of specific receptor-expressing target neurons (discussed above) will clarify these possibilities.

Nonetheless, our data outline how neuropeptides from a single group of neurons can functionally reconfigure different receptor-expressing neurons in a peptide dose-dependent manner. The existence of multiple receptors is important for diversifying neuromodulator targets. In vertebrates, D1 and D2 dopamine receptors label largely nonoverlapping subpopulations of medium spiny neurons (Gerfen et al., 1990; Gong et al., 2007), which play complementary roles in motion control (Jin et al., 2014; Geddes et al., 2018). In *Drosophila*, different dopamine receptors play distinct roles in both innate (Zhang et al., 2016; Sayin et al., 2019) and learned (Handler et al., 2019) behaviors, at least in part by activating different downstream signaling cascades (Handler et al., 2019). As for neuropeptides, diuretic hormone 44 (Dh44) released from the glucose-sensing neurons in the central brain of *Drosophila* acts on two distinct downstream target neurons labeled by expression of two different receptors, *Dh44-R1* (in downstream neurons) and *Dh44-R2* (in gut cells; Dus et al., 2015). These two cell types coordinate starvation-induced behavioral and physiological changes. Collectively, these examples depict a motif whereby multiple receptors of a neuromodulator define functionally distinct downstream circuits. Our results indicate that different downstream targets of aggression-promoting Tk-GAL4^FruM^ neurons are recruited depending on the peptide level from a single cluster of neurons, contributing to distinct aspects of behavioral escalation.

All six mature peptides (DTK1–DTK6) generated from the tachykinin prepropeptide can activate *TkR99D* (Birse et al., 2006; Jiang et al., 2013), whereas *TkR86C*, whose preferred ligand is natalisin (Jiang et al., 2013), can be activated only by a high concentration of DTK6 (Poels et al., 2009; Jiang et al., 2013). Although these pharmacological characteristics appear somewhat inconsistent with our observation that *TkR99D*-expressing neurons could be activated only when tachykinin was overexpressed, effective concentration of neuropeptides on target neurons can depend on how the source and receptors are positioned. *TkR86C*-expressing neurons may receive tachykinin in or near the synaptic clefts, which can facilitate transient increase of peptide concentration to a level sufficient to engage *TkR86C*.

Naturalistic conditions that induce a high level of tachykinin expression in Tk-GAL4^FruM^ neurons remain unknown. In mice, one of the two tachykinin-encoding genes (*Tac2*) is upregulated after social isolation stress (Zelikowsky et al., 2018). Previous anatomic studies suggested that Tk-GAL4^FruM^ neurons may be capable of integrating incoming chemosensory information (Yu et al., 2010), but no synaptic inputs have been identified yet. One possibility is that Tk-GAL4^FruM^ neurons serve as a coincidence detector of multiple factors that collectively promote aggression, such as social isolation (Wang et al., 2008), increased male density (Wang and Anderson, 2010), and mating condition (Yuan et al., 2014). Identification of behavioral experiences or physiological conditions that cause increased tachykinin release from Tk-GAL4^FruM^ neurons will be necessary for understanding the ethological functions of tachykinin receptor-expressing neurons.

Tachykinins constitute an evolutionarily conserved family of neuropeptides (Severini et al., 2002; Nässel et al., 2019). It is intriguing that tachykinins are known to control aggressive behaviors in several mammalian species (Katsouni et al., 2009; Zelikowsky et al., 2018). Whereas vertebrate tachykinins (such as substance P) are considered excitatory neuropeptides (Phillis and Limacher, 1974; Jan and Jan, 1982), *Drosophila* tachykinin is known to act as an inhibitory modulator (Ignell et al., 2009; Ko et al., 2015; Lee et al., 2021). Our study demonstrates that *Drosophila* tachykinin can also act as an excitatory neuromodulator. Consistently, both *TkR86C* and *TkR99D* receptors transfected in a cell culture caused intracellular calcium increase on application of tachykinin (Johnson et al., 2003; Birse et al., 2006; Poels et al., 2009; Jiang et al., 2013). How can one neuropeptide species act as both an excitatory and an inhibitory neuromodulator? One possibility is that *Drosophila* tachykinin receptors may couple with excitatory or inhibitory G-proteins in different neuronal populations. Alternatively, different neuropeptides may have different pharmacological impacts on the receptors. Neuromodulatory cells in different microcircuits may release distinct mixtures of mature neuropeptides, which could elicit circuit-specific physiological effects. Specifically, it is possible that *TkR86C-*expressing neurons can be additionally modulated by natalisin-releasing neurons, which project widely across the adult brain (Jiang et al., 2013). Finally, tachykinin receptors can engage multiple intracellular signaling cascades. Future investigations on the molecular mechanisms of tachykinergic neuromodulation will help predict the physiological and behavioral effects of pharmacological substances that are designed to target specific receptor-expressing neurons (Holmes et al., 2003; Griebel and Holsboer, 2012).

A neuromodulator can affect circuits and behavior in a functionally distinct way from a coexpressed neurotransmitter, as shown both in flies (Sherer et al., 2020) and in mice (Chen et al., 2019; Zell et al., 2020). Because neuromodulators (especially neuropeptides) may communicate with receptor-expressing neurons extrasynaptically, the connectome by itself may not fully reveal all the physiologically and behaviorally relevant functional relationships among neurons. The expression profiles of neuromodulator receptors (coined the “chemoconnectome”; Deng et al., 2019)) in these aggression-controlling neuromodulatory cells may provide an insight into their functional connectivity.

How tachykininergic systems interface with other aggression-controlling peptidergic systems, such as neuropeptide F (Dierick and Greenspan, 2007) and Drosulfakinin (Agrawal et al., 2020; Wu et al., 2020) or biogenic amine neuromodulators (Dierick and Greenspan, 2007; Hoyer et al., 2008; Zhou et al., 2008; Certel et al., 2010; Alekseyenko et al., 2013, 2014, 2019; Andrews et al., 2014; Watanabe et al., 2017), remains an important question to be resolved. To delineate the contributions of each neuromodulator-releasing neuronal group, it will be critical to identify the behavioral context in which each population is engaged. Each neuromodulator may represent a specific internal or external condition that helps the animal weigh the costs and benefits of fighting. In the case of the tachykininergic system, characterization of the neural inputs into Tk-GAL4^FruM^ neurons and determinants of tachykinin release amount will help us understand which aspects of strategic decision-making are mediated by this population of neurons and how tachykinins serve as a molecular actuator of the consequential behavioral choices.
